# Inherited determinants of Crohn's disease and ulcerative colitis phenotypes: a genetic association study

**DOI:** 10.1016/S0140-6736(15)00465-1

**Published:** 2016-01-09

**Authors:** Isabelle Cleynen, Gabrielle Boucher, Luke Jostins, L Philip Schumm, Sebastian Zeissig, Tariq Ahmad, Vibeke Andersen, Jane M Andrews, Vito Annese, Stephan Brand, Steven R Brant, Judy H Cho, Mark J Daly, Marla Dubinsky, Richard H Duerr, Lynnette R Ferguson, Andre Franke, Richard B Gearry, Philippe Goyette, Hakon Hakonarson, Jonas Halfvarson, Johannes R Hov, Hailang Huang, Nicholas A Kennedy, Limas Kupcinskas, Ian C Lawrance, James C Lee, Jack Satsangi, Stephan Schreiber, Emilie Théâtre, Andrea E van der Meulen-de Jong, Rinse K Weersma, David C Wilson, International Inflammatory Bowel Disease Genetics Consortium, Miles Parkes, Severine Vermeire, John D Rioux, John Mansfield, Mark S Silverberg, Graham Radford-Smith, Dermot P B McGovern, Jeffrey C Barrett, Charlie W Lees

**Affiliations:** a Wellcome Trust Sanger Institute, Wellcome Trust Genome Campus, Hinxton, Cambridge, UK; b Department of Clinical and Experimental Medicine, TARGID, KU Leuven, Leuven, Belgium; c Université de Montréal and the Montreal Heart Institute, Research Center, Montréal, Québec, Canada; d Wellcome Trust Centre for Human Genetics, University of Oxford, Oxford, UK; e Christ Church, University of Oxford, St Aldates, UK; f Department of Public Health Sciences, University of Chicago, Chicago, IL, USA; g Department for General Internal Medicine, Christian-Albrechts-University, Kiel, Germany; h Institute of Clinical Molecular Biology, Christian-Albrechts-University, Kiel, Germany; i Department for General Internal Medicine, Christian-Albrechts-University, Kiel, Germany; j Peninsula College of Medicine and Dentistry, Exeter, UK; k Medical Department, Viborg Regional Hospital, Viborg, Denmark; l Hospital of Southern Jutland Aabenraa, Aabenraa, Denmark; m Inflammatory Bowel Disease Service, Department of Gastroenterology and Hepatology, Royal Adelaide Hospital, Adelaide, Australia; n School of Medicine, University of Adelaide, Adelaide, Australia; o Unit of Gastroenterology, Istituto di Ricovero e Cura a Carattere Scientifico-Casa Sollievo della Sofferenza (IRCCS-CSS) Hospital, San Giovanni Rotondo, Italy; p Azienda Ospedaliero Universitaria (AOU) Careggi, Unit of Gastroenterology SOD2, Florence, Italy; q Department of Medicine II, University Hospital Munich-Grosshadern, Ludwig-Maximilians-University, Munich, Germany; r Meyerhoff Inflammatory Bowel Disease Center, Department of Medicine, School of Medicine, Johns Hopkins University, Baltimore, MD, USA; s Department of Epidemiology, Bloomberg School of Public Health, Johns Hopkins University, Baltimore, MD, USA; t Department of Genetics, Yale School of Medicine, New Haven, CT, USA; u Broad Institute of MIT and Harvard, Cambridge, MA, USA; v Department of Pediatrics, Cedars Sinai Medical Center, Los Angeles, CA, USA; w Division of Gastroenterology, Hepatology and Nutrition, Department of Medicine, University of Pittsburgh School of Medicine, Pittsburgh, PA, USA; x Department of Human Genetics, University of Pittsburgh Graduate School of Public Health, Pittsburgh, PA, USA; y School of Medical Sciences, Faculty of Medical and Health Sciences, University of Auckland, Auckland, New Zealand; z Department of Medicine, University of Otago, Christchurch, New Zealand; aa Department of Gastroenterology, Christchurch Hospital, Christchurch, New Zealand; ab Center for Applied Genomics, Children's Hospital of Philadelphia, Philadelphia, PA, USA; ac Department of Gastroenterology, Faculty of Medicine and Health, Örebro University, Sweden; ad School of Health and Medical Sciences, Örebro University, Örebro, Sweden; ae Norwegian PSC Research Center, Research Insitute of Internal Medicine and Department of Transplantation Medicine, Oslo University Hospital and University of Oslo, Oslo, Norway; af Gastrointestinal Unit, Institute of Genetics and Molecular Medicine, University of Edinburgh, Edinburgh, UK; ag Child Life and Health, University of Edinburgh, Edinburgh, UK; ah Department of Gastroenterology, Lithuanian University of Health Sciences, Kaunas, Lithuania; ai Centre for Inflammatory Bowel Diseases, Saint John of God Hospital, Subiaco WA and School of Medicine and Pharmacology, University of Western Australia, Harry Perkins Institute for Medical Research, Murdoch, WA, Australia; aj Inflammatory Bowel Disease Research Group, Addenbrooke's Hospital, University of Cambridge, Cambridge, UK; ak Unit of Animal Genomics, Groupe Interdisciplinaire de Genoproteomique Appliquee (GIGA-R) and Faculty of Veterinary Medicine, University of Liege, Liege, Belgium; al Division of Gastroenterology, Centre Hospitalier Universitaire, Universite de Liege, Liege, Belgium; am Department of Gastroenterology and Hepatology, Leiden University Medical Center, Leiden, Netherlands; an Department of Gastroenterology and Hepatology, University of Groningen and University Medical Center Groningen, Groningen, Netherlands; ao Royal Hospital for Sick Children, Paediatric Gastroenterology and Nutrition, Glasgow, UK; ap Division of Gastroenterology, University Hospital Gasthuisberg, Leuven, Belgium; aq Institute of Human Genetics, Newcastle University, Newcastle upon Tyne, UK; ar Mount Sinai Hospital Inflammatory Bowel Disease Centre, University of Toronto, Toronto, ON, Canada; as Inflammatory Bowel Diseases, Genetics and Computational Biology, Queensland Institute of Medical Research, Brisbane, Australia; at Department of Gastroenterology, Royal Brisbane and Women's Hospital, and School of Medicine, University of Queensland, Brisbane, Australia; au F Widjaja Foundation Inflammatory Bowel and Immunobiology Research Institute, Cedars-Sinai Medical Center, Los Angeles, CA, USA

## Abstract

**Background:**

Crohn's disease and ulcerative colitis are the two major forms of inflammatory bowel disease; treatment strategies have historically been determined by this binary categorisation. Genetic studies have identified 163 susceptibility loci for inflammatory bowel disease, mostly shared between Crohn's disease and ulcerative colitis. We undertook the largest genotype association study, to date, in widely used clinical subphenotypes of inflammatory bowel disease with the goal of further understanding the biological relations between diseases.

**Methods:**

This study included patients from 49 centres in 16 countries in Europe, North America, and Australasia. We applied the Montreal classification system of inflammatory bowel disease subphenotypes to 34 819 patients (19 713 with Crohn's disease, 14 683 with ulcerative colitis) genotyped on the Immunochip array. We tested for genotype–phenotype associations across 156 154 genetic variants. We generated genetic risk scores by combining information from all known inflammatory bowel disease associations to summarise the total load of genetic risk for a particular phenotype. We used these risk scores to test the hypothesis that colonic Crohn's disease, ileal Crohn's disease, and ulcerative colitis are all genetically distinct from each other, and to attempt to identify patients with a mismatch between clinical diagnosis and genetic risk profile.

**Findings:**

After quality control, the primary analysis included 29 838 patients (16 902 with Crohn's disease, 12 597 with ulcerative colitis). Three loci (*NOD2, MHC*, and *MST1* 3p21) were associated with subphenotypes of inflammatory bowel disease, mainly disease location (essentially fixed over time; median follow-up of 10·5 years). Little or no genetic association with disease behaviour (which changed dramatically over time) remained after conditioning on disease location and age at onset. The genetic risk score representing all known risk alleles for inflammatory bowel disease showed strong association with disease subphenotype (p=1·65 × 10^−78^), even after exclusion of *NOD2, MHC*, and 3p21 (p=9·23 × 10^−18^). Predictive models based on the genetic risk score strongly distinguished colonic from ileal Crohn's disease. Our genetic risk score could also identify a small number of patients with discrepant genetic risk profiles who were significantly more likely to have a revised diagnosis after follow-up (p=6·8 × 10^−4^).

**Interpretation:**

Our data support a continuum of disorders within inflammatory bowel disease, much better explained by three groups (ileal Crohn's disease, colonic Crohn's disease, and ulcerative colitis) than by Crohn's disease and ulcerative colitis as currently defined. Disease location is an intrinsic aspect of a patient's disease, in part genetically determined, and the major driver to changes in disease behaviour over time.

**Funding:**

International Inflammatory Bowel Disease Genetics Consortium members funding sources (see Acknowledgments for full list).

## Introduction

Crohn's disease and ulcerative colitis, the two major forms of inflammatory bowel disease, affect about one in 200 people in developed countries, with a rising incidence and prevalence in developing countries.^1^ Many patients with inflammatory bowel disease have a lifetime of debilitating physical symptoms (eg, urgent diarrhoea, rectal bleeding, vomiting, anorexia, and lethargy), which frequently lead to poor psychosocial wellbeing with wide ranging consequences for academic attainment, employment, relationships, and sexual health.^2^ Furthermore, the financial costs of inflammatory bowel disease are substantial and are estimated at more than US$2·2 billion per year in the USA alone.^3^

Inflammatory bowel disease is characterised by an exaggerated mucosal immune response to luminal gut contents in genetically susceptible individuals.^4^ In Crohn's disease, inflammation can occur in any part of the gastrointestinal tract, whereas ulcerative colitis is typically confined to the colon. The universally adopted Montreal classification distinguishes clinical subphenotypes in Crohn's disease by disease location and behaviour, and age of onset, and in ulcerative colitis by disease extent and age of onset.^5^, ^6^, ^7^, ^8^, ^9^, ^10^, ^11^ Molecular studies have suggested that ileal and colonic Crohn's disease are distinct entities because variants in *NOD2* are associated with small bowel disease and *HLA* alleles with colonic disease.^12^, ^13^, ^14^, ^15^, ^16^, ^17^, ^18^, ^19^ However, current recommendations do not advocate the use of these established markers in making treatment decisions, nor for choosing patients for clinical trials.^20^, ^21^, ^22^, ^23^, ^24^, ^25^, ^26^, ^27^, ^28^, ^29^ The natural history and clinical course of inflammatory bowel disease is very heterogeneous: up to 20% of patients with ulcerative colitis need colectomy for medically refractory disease, and more than 50% of patients with Crohn's disease need surgery within 10 years of diagnosis;^30^ however, up to 50% of patients with ulcerative colitis and 30% with Crohn's disease will have a fairly indolent disease course without the need for immunosuppression or surgery.^31^, ^32^

**Research in context**

**Evidence before this study**

We searched PubMed for genotype–phenotype association studies in inflammatory bowel disease, published between Jan 1, 1996, and Oct 17, 2014, with the search terms “inflammatory bowel disease” AND “genotype phenotype” AND “genetic association” AND (“disease course” or “disease extent” or “location behaviour”). We found 31 studies with sample sizes of 66 to 2804 patients, and studying between one and 163 genetic variants. Most of these studies implicated *NOD2* and *HLA* in subphenotypes of inflammatory bowel disease. Many studies, especially the early ones, studied only *NOD2* and *HLA*, and none included genetic variants not previously implicated in risk for inflammatory bowel disease.

**Added value of this study**

This study is the largest genotype–phenotype study of inflammatory bowel disease by at least a factor of ten, and is among the largest studies of genetic determinants of clinical subphenotypes of any complex disease. We have refined the known associations (for instance, the effect of *NOD2* on disease behaviour is entirely driven by its association with disease location) and discovered one new associated locus (3p21/*MST1* with age at diagnosis). We have explored the genetic relations between subtypes of inflammatory bowel disease with genetic risk scores for the first time, and have shown that ileal and colonic Crohn's disease are at least as genetically distinct from each other as they are from ulcerative colitis.

**Implications of all the available evidence**

Established genetic factors can only explain a small fraction of the variability in subphenotype of inflammatory bowel disease, but genetic risk scores that capture all this information could be used to identify misdiagnosed patients. Future translational and clinical research should move away from a binary classification of inflammatory bowel disease into ulcerative colitis and Crohn's disease, instead considering ileal and colonic Crohn's disease as separate disease entities.

Inflammatory bowel disease has been at the vanguard of progress in understanding the genetic framework of complex diseases, with 163 susceptibility loci identified so far.^33^ Most inflammatory bowel disease loci confer risk of both ulcerative colitis and Crohn's disease, but typically show distinct effect sizes in the two disorders. These findings suggest that genetic variation might define molecular subtypes independent of traditional and clinically defined diagnostic entities, allowing new insights into the molecular basis of these subphenotypes.

Our international study of around 30 000 patients with inflammatory bowel disease genotyped by microarray is the largest genotype–subphenotype study in the disease done so far. We have used genetic risk scores to study genetic heterogeneity underpinning the natural history of inflammatory bowel disease. This analysis rejects the current binary classification of Crohn's disease and ulcerative colitis as distinct and homogeneous clinical entities in favour of a continuum of illness better fit by a three-category model (ie, ileal Crohn's disease, colonic Crohn's disease, and ulcerative colitis). We show that these risk scores have clinical potential (although they are currently only weak predictors), and believe they might have widespread applicability in other diseases.

## Methods

### Study design and patients

We acquired phenotype data for 34 819 patients, including 19 713 with Crohn's disease and 14 683 with ulcerative colitis. The cohort included all patients in different centres over the years who had inflammatory bowel disease as per Lennard-Jones' criteria.^34^ All inclusion criteria are included in appendix A. After quality control (appendix A), the primary analysis included 29 838 patients (16 902 with Crohn's disease, 12 597 with ulcerative colitis, 255 with indeterminate colitis, and 84 missing an exact diagnosis). This study includes patients from population-based registries, and secondary and tertiary-referral centres at 49 sites in 16 countries in Europe, North America, and Australasia, most of which have been previously described (appendix B).^33^ Confirmation of diagnosis of inflammatory bowel disease and assignment of clinical subphenotypes were done by clinicians specialising in inflammatory bowel disease or trained phenotypers through case note reviews of clinical, radiological, histopathological, and endoscopic reports, and classified per the Montreal classification criteria (see appendix A for details).^8^, ^9^ For behaviour and surgery in Crohn's disease, and colectomy in ulcerative colitis, Kaplan-Meier survival curves, stratified by location of Crohn's disease and extent of ulcerative colitis, were drawn to estimate time to first event (see appendix A for details).

The ethical boards of each separate recruiting centre approved the study. All patients included in this study gave written informed consent.

### Procedures

All cases were genotyped with the Immunochip array (Illumina, San Diego, CA, USA; appendix B) as previously described.^33^ Briefly, the Immunochip is a 195 806-polymorphism genotyping platform comprising variants identified from association studies of immune-related disorders including Crohn's disease and ulcerative colitis. Extensive quality control was performed on the dataset (appendix A), leaving 29 838 cases and 156 154 markers available for analyses. Variants in the MHC, including 23 *HLA* alleles that have been implicated in inflammatory bowel disease, were imputed as described in appendix A.^35^

All association tests were done on all genotyped variants, conditional on the first five principal components to account for population structure. Age of onset was analysed for Crohn's disease and ulcerative colitis separately and then meta-analysed; time to surgery was analysed with parametric survival-time regression models; and upper gastrointestinal involvement and perianal disease were analysed with binary logistic regression (see appendix A). For multicategory phenotypes (disease location, behaviour, and extent) we used model selection to pick the most appropriate genetic model for the phenotype (appendix A). The model selection indicated a multinomial model for Crohn's disease location (ie, three unordered categories), an ordinal logistic model for Crohn's disease behaviour (three ordered categories, B3 penetrating>B2 stricturing>B1 inflammatory), and a binary model for disease extent of ulcerative colitis (two categories: E3 extensive disease *vs* E2 left-sided disease and E1 proctitis). To distinguish direct associations from indirect (ie, driven by an association with a correlated phenotype), we also adjusted all regression models for the other phenotypes (age of onset, location, and behaviour for Crohn's disease; age of onset and disease extent for ulcerative colitis). Genome-wide significance (p<5 × 10^−08^) was required for individual single nucleotide polymorphisms (SNPs) and *HLA* types.

All signals that showed suggestive association (p<1 × 10^−5^) with any of the disease subphenotypes were assessed in an independent cohort genotyped on a range of different genome-wide association study (GWAS) chips. These samples have also undergone rigorous quality control and imputation.^33^ Phenotype data for an additional and independent 2453 patients with Crohn's disease and 3729 patients with ulcerative colitis were available for these analyses. See appendix A for additional information about the replication cohort.

To learn about the relative phenotypic variance explained by different risk factors in adult inflammatory bowel disease, we fitted a model to predict Crohn's disease location that included both demographic predictors (smoking status, age at diagnosis, and year) and genetic predictors (SNPs at *NOD2, MST1*, and the *HLA* cluster as well as the genetic risk score). Variance explained on the logit scale by each predictor was calculated with the McKelvey–Zavoina pseudo R^2^. Centres with a high proportion (>60%) of missing data for smoking status were removed. To reduce the effect of changes in clinical practice and smoking rates, only patients born between 1955 and 1985 were included.

In addition to looking at single SNPs, we also combined information from 193 SNPs and 23 *HLA* types previously associated with inflammatory bowel disease to generate genetic risk scores (appendix A), which provide better predictive accuracy than individual SNPs. To assess classification accuracy, we re-ran the risk score analyses with a cross-validation strategy, in which models were fitted in non-UK origin samples and assessed by how well they classified UK samples.

To assess if the risk score can be used to identify misclassified patients, we selected 97 outlier patients that fell in the extreme tail of the scores for the opposite phenotype (log Crohn's disease versus ulcerative colitis [CD *vs* UC] score ≤–2 for Crohn's disease outliers and log CD *vs* UC score ≥2 for ulcerative colitis outliers), as well as 95 randomly selected cases with non-outlier scores matched by recruitment centre. Clinicians from each centre were then asked to re-phenotype both outlier and non-outlier patients in a masked fashion. The CD versus UC risk score was chosen for this experiment because it had the strongest association with Crohn's disease location and behaviour.

### Statistical analysis

The median effect size of known inflammatory bowel disease risk variants^33^ was about OR 1·1, with a median minor allele frequency of roughly 30%. The sample size of our study gave us high power to detect an effect of equivalent magnitude of Crohn's disease location (power of 67% for ileal *vs* non-ileal disease), Crohn's disease behaviour (94% for complicated *vs* non-complicated disease) and ulcerative colitis disease extent (84% for extensive *vs* non-extensive disease) at genome-wide significance. Binary and linear genotype–phenotype analyses were done with PLINK version 1.07,^36^ and multinomial and ordinal regression with a custom program, Trinculo version 0.4 (appendix A). Survival analysis and risk prediction were done with R-2.15.1 using the packages “survival” and “Mangrove”,^37^ respectively. Data handling and plotting was done with R.

### Role of the funding source

The funders of the study had no role in study design, data collection, data analysis, data interpretation, or writing of the report. All authors had full access to all the data in the study and had final responsibility for the decision to submit for publication.

## Results

**Figure 1:**
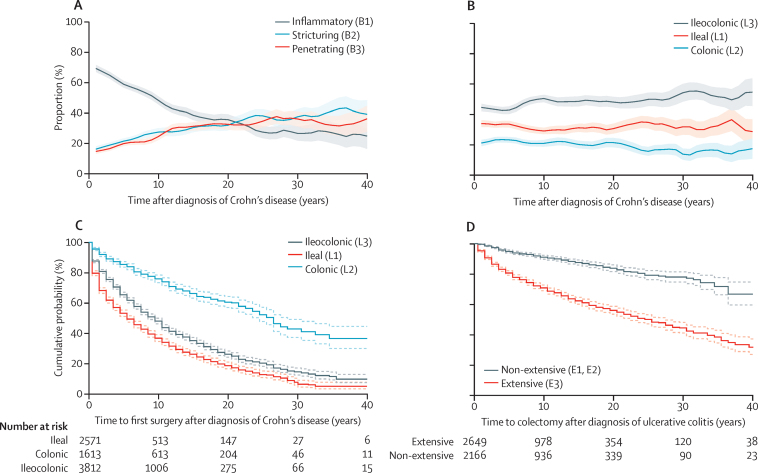
Evolution of clinical subphenotypes (A) Proportion of patients with Crohn's disease who have inflammatory (Montreal classification B1), stricturing (B2), or penetrating (B3) disease over time from diagnosis to most recent follow-up. (B) Proportion of patients with Crohn's disease who have ileal (L1), colonic (L2), or ileocolonic (L3) disease over time from diagnosis to most recent follow-up. (C) Survival plot of time from diagnosis of Crohn's disease to resectional surgery stratified by disease location. (D) Survival plot of time from diagnosis of ulcerative colitis to colectomy stratified by disease extent (extensive disease, E3; non-extensive disease, E1 and E2).

Our primary analyses were done on matched genotype and phenotype data from 29 838 patients of European ancestry (appendix A) with inflammatory bowel disease (16 902 with Crohn's disease, 12 597 with ulcerative colitis; table 1) with a total of 217 195 patient-years of follow-up (median per patient: 11 years for Crohn's disease and 10 years for ulcerative colitis). Demographic features of the study population agreed with previously published results: patients with Crohn's disease were more likely to be younger at diagnosis, female, smokers, and have affected family members than were patients with ulcerative colitis (table 1). Extensive disease was more common in those diagnosed at a younger age in both Crohn's disease and ulcerative colitis, whereas disease behaviour was relatively unaffected by age at diagnosis (appendix A). Reaffirming the progressive nature of Crohn's disease, the proportion of patients with stricturing (B2) or penetrating (B3) disease increased from less than 30% (n/N) at diagnosis to 43% (n/N) at 5 years, 56% (n/N) at 10 years, and 74% (n/N) at 30 years (figure 1, which shows the progression in B1, B2, and B3 disease individually [smoothed estimates over intervals]). By contrast, disease location showed little variation during the same period (figure 1). With the exception of the population-based cohorts from Scandinavia, survival analyses of time to development of complicated disease (B2, B3) or first surgery in Crohn's disease were highly consistent across the different countries of origin despite different health-care systems and methods of sampling (appendix A). In Crohn's disease, time from diagnosis to progression (complicated disease or surgical intervention) was significantly shorter in purely ileal (L1) compared with ileocolonic (L3) or colonic (L2) disease (p<10^−100^; figure 1; appendix A). Overall, 7257 (52%) of 13 862 patients with Crohn's disease had undergone surgery by the time of last follow-up. In ulcerative colitis, in which the overall rate of colectomy was 22% 10 years after diagnosis, time to surgery was shorter in patients with extensive disease (E3) than in those with left-sided disease (E2) or proctitis (E1; p=8 × 10^−84^; figure 1).

**Figure 2:**
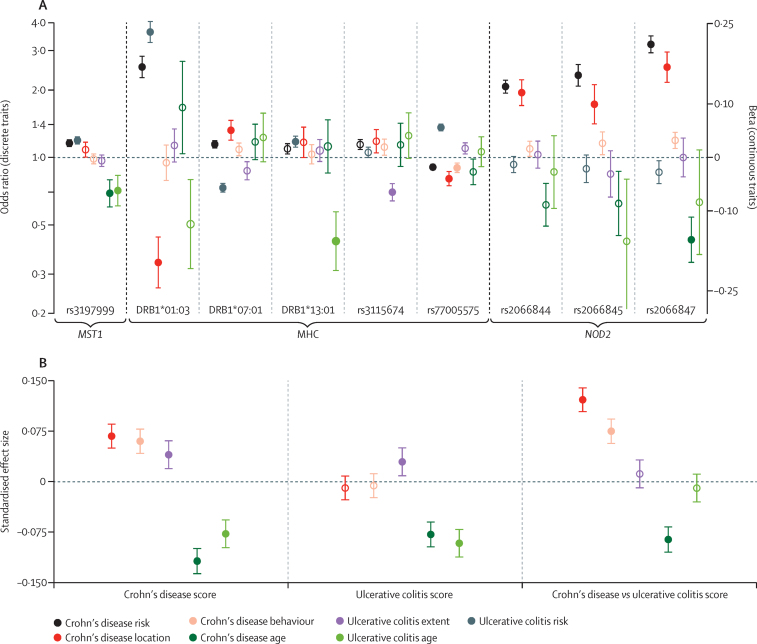
Effect of single nucleotide polymorphisms, *HLA* alleles, and polygenic risk scores on phenotypes of inflammatory bowel disease (A) Effect sizes for genotype–phenotype associations for risk of Crohn's disease and ulcerative colitis (odds ratio relative to controls), Crohn's disease location (odds ratio of ileal *vs* colonic disease), Crohn's disease behaviour (proportional odds ratio), disease extent of ulcerative colitis (odds ratio of extensive *vs* non-extensive disease), and age at diagnosis (linear coefficients) for *MST1*, MHC, and *NOD2* variants. All effect sizes are per allele, and are adjusted for associations with correlated phenotypes by including them as additional predictors in the regression model, along with principal components to control for stratification. See appendix A for more details on these regression models. Genome-wide significant associations are depicted by filled circles, and error bars depict 95% CIs. (B) Effect sizes of genetic risk scores for disease location, disease behaviour, and age at diagnosis including all 163 susceptibility loci. Effect sizes are calculated by linear regression of the risk score against the phenotype, adjusted for the effect of the other phenotypes and for principal components, and error bars depict 95% CIs. Filled circles represent effects that are significant after correcting for 15 phenotype-score combinations (p<0·003). Effect sizes are measured on scales standardised to unit variance (and thus represent the number of standard deviations that the mean phenotype increases by per standard deviation increase in the risk score).

We tested genetic variants for association with age at diagnosis and time to surgery in all patients with inflammatory bowel disease; disease location and behaviour in Crohn's disease; and disease extent in ulcerative colitis (table 2 and table 3). Across all analyses, three loci achieved genome-wide significance (p<5 × 10^−8^): 3p21 (*MST1), NOD2*, and the MHC. No additional signals were noted after replication of suggestive loci (p<1 × 10^−5^) in an independent GWAS cohort (appendix B). Although *NOD2* was strongly associated with Crohn's disease location, behaviour, and age at diagnosis, adjustment for the other phenotypes showed that the association of *NOD2* with behaviour was driven almost entirely by its phenotypic correlation with location and age at diagnosis (figure 2).

We noted complex and correlated *HLA* signals for susceptibility to inflammatory bowel disease overall and age at onset, as well as Crohn's disease location and behaviour, extent of ulcerative colitis, and surgery (figure 2; appendix A; appendix B). In-depth analysis of the MHC region including classical *HLA* alleles showed that the strongest signal for disease location was a colonic association with HLA-DRB1*01:03 (p=1·47 × 10^−23^; ileal *vs* colonic odds ratio [OR] 0·32, 95% CI 0·29–0·41; ileocolonic *vs* colonic OR 0·47, 0·39–0·57), which is also the strongest shared risk allele for Crohn's disease and ulcerative colitis,^35^ followed by HLA-DRB1*07:01 (figure 2; appendix A). rs77005575 was independently associated with Crohn's disease behaviour (p=1·56 × 10^−09^; figure 2; appendix A). Notably, alleles associated with susceptibility to ulcerative colitis were better predictors of colonic disease location in Crohn's disease than alleles associated with susceptibility to Crohn's disease (appendix A). The top signal for extent of ulcerative colitis was rs3115674 (p=5·11 × 10^−17^ OR 0·70, 0·64–0·76; appendix A), which correlates with HLA-B*08 (R^2^=0·66), found mostly on the ancestral 8.1 *HLA* haplotype. HLA-DRB1*13:01 was the top signal for age at diagnosis of ulcerative colitis (3·50 × 10^−09^; figure 2; appendix A).

On the basis of sample size, our primary analysis had similar power to detect associations to disease location (ileal *vs* colonic) as the first International Inflammatory Bowel Disease Genetics Consortium (IIBDGC) meta-analysis on Crohn's disease.^38^ However, with the exception of *NOD2,* MHC, and *MST1,* we do not report significant associations between subphenotypes and individual SNPs, including those robustly associated with disease susceptibility. We noted, however, that many known risk loci showed nominal evidence for association to a range of subphenotypes, so we posited that genetic risk scores representing the combined effect of many individually weak signals might be a more powerful approach to study the genetic underpinnings of subphenotypes for inflammatory bowel disease. We calculated different inflammatory bowel disease risk scores constructed from all available data (strength and direction of association) on the lead SNPs from each of the 163 known inflammatory bowel disease susceptibility loci. Although all of the risk scores were associated with Crohn's disease and ulcerative colitis subphenotypes, the most powerful score used the differences between Crohn's disease and ulcerative colitis (CD *vs* UC score; figure 2). Importantly, this CD versus UC score retained significance even after *NOD2,* MHC, and *MST1* were removed (appendix A), lending support to the notion that the genetic risk score offers more information about the genetic substructure of inflammatory bowel disease than individual SNP associations alone. The strongest correlations in our study were between the CD versus UC risk score and Crohn's disease location and behaviour (figure 2**;** p=1·65 × 10^−78^, or p=9·23 × 10^−18^ after genome-wide significant loci were removed). Risk scores that incorporated imputed HLA types that have been implicated in risk for inflammatory bowel disease significantly improved the genetic risk scores compared with those using SNPs only (appendix A).

**Figure 3:**
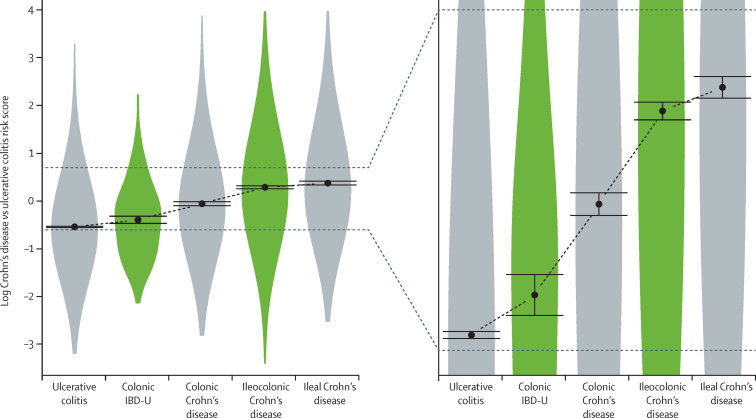
Violin plot showing the genetic substructure of inflammatory bowel disease location The violin represents the range of the log CD versus UC score for the indicated subphenotype (calculated with the R package “vioplot”), with dots representing the mean of that group and error bars the 95% CIs. Although the effects are small compared with the variation within groups, the mean effects can still be measured accurately (right side of the figure). It can be seen on this figure that the Crohn's disease versus ulcerative colitis (CD *vs* UC) risk score placed colonic Crohn's disease between ileal Crohn's disease and ulcerative colitis. The plot also shows the positioning of the intermediate phenotypes (ileocolonic Crohn's disease and inflammatory bowel disease unclassified [IBD-U]) in between ileal and colonic Crohn's disease, and ulcerative colitis and colonic Crohn's disease, respectively.

Having shown the genetic risk score to be a useful measurement of inflammatory bowel disease subphenotype, we used it to study the genetic relation between ileal Crohn's disease, colonic Crohn's disease, and ulcerative colitis. The CD versus UC risk score placed colonic Crohn's disease between ileal Crohn's disease and ulcerative colitis (figure 3). Several other risk scores supported this relation, and although partly driven by the highly location-specific *NOD2* variants, a risk score with *NOD2* removed showed a similar pattern (appendix A). Additionally, statistical model selection across SNPs and *HLA* types strongly favoured a model in which colonic Crohn's disease is intermediate between ileal Crohn's disease and ulcerative colitis over one that grouped both Crohn's disease subphenotypes as a single category (appendix A). To test whether this finding extends to other subtypes of inflammatory bowel disease, we applied the genetic risk score to two intermediate forms of the disease: ileocolonic Crohn's disease (L3), in which the disease affects both small and large bowel, and colonic inflammatory bowel disease unclassified, in which the clinical and histological appearances are indistinguishable between Crohn's disease and ulcerative colitis. The CD versus UC score placed ileocolonic Crohn's disease as intermediate between ileal (L1) and colonic (L2) Crohn's disease, and colonic inflammatory bowel disease unclassified between ulcerative colitis and colonic Crohn's disease (figure 3).

Despite the statistical significance of the associations between genetic risk score and subphenotype, the small effect sizes translated into fairly low predictive accuracy when tested by cross-validation (appendix A). The risk score that was most significantly associated with location of Crohn's disease in the primary analysis (CD *vs* UC) gave an area under the receiver-operating characteristic (ROC) curve of only 0·60 (95% CI 0·57–0·63) for distinguishing between colonic (L2) and ileal (L1) Crohn's disease in cross-validation, and even a specifically constructed ileal versus colonic score achieved an area under the curve of only 0·63 (0·59–0·66; appendix A). Comparison of the clinical characteristics of patients with Crohn's disease that fell into the extreme tails of the genetic risk score (low: log[CD *vs* UC] ≤–2; high: log[CD *vs* UC] ≥3) showed significant differences in disease location and behaviour (appendix A), suggesting that although a genetic risk score might not be able to classify all patients, it could be informative at the extremes.

Overall, we can only explain a little of the phenotypic variance in the adult population with either classical or genetic predictors. The combination of smoking and the strongest genetic predictors explains only 6·8% of the variance for disease location in Crohn's disease (table 4 and table 5), and 1·1% for disease extent in ulcerative colitis (table 5).

**Figure 4:**
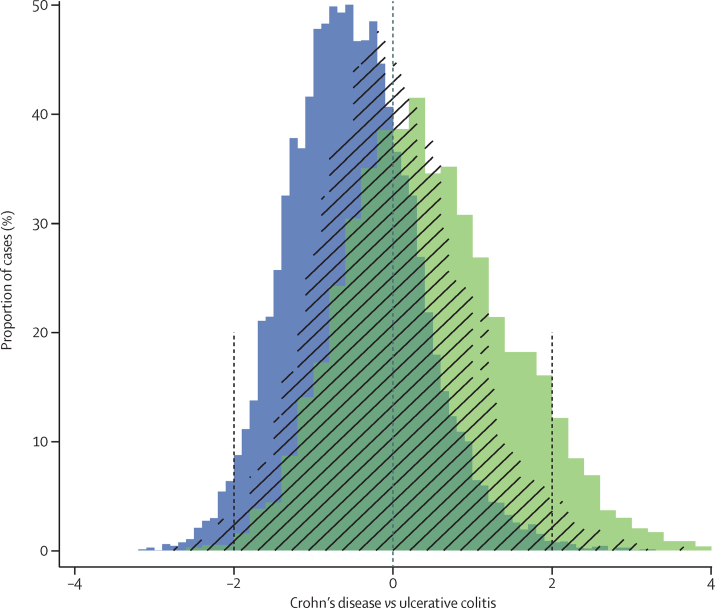
Histograms of Crohn's disease versus ulcerative colitis (CD *vs* UC) genetic risk score in patients with inflammatory bowel disease Risk scores created from the 163 known inflammatory bowel disease risk loci with per-locus contributions estimated to maximally distinguish all Crohn's disease from ulcerative colitis. Distributions of ulcerative colitis samples are shown in blue, ileal Crohn's disease samples in green, and colonic Crohn's disease with hatched lines (middle area in dark green shows overlap of blue and green distributions). The overlap of all three distributions shows the shared genetic aetiology of inflammatory bowel disease, and the intermediate position of colonic Crohn's disease between ulcerative colitis and ileal Crohn's disease shows that it is genetically distinct from the others. Vertical dashed lines show boundaries for outlier analysis: ulcerative colitis cases above 2 were selected as being likely Crohn's disease and Crohn's disease cases below −2 as likely ulcerative colitis.

To assess the possible clinical usefulness of the genetic risk score, we reassessed the cases of Crohn's disease with a low CD versus UC risk score (more ulcerative colitis-like), and cases of ulcerative colitis with a high CD versus UC risk score (more Crohn's disease-like; figure 4). Masked re-phenotyping of these cases raised doubts about the original diagnosis in 27% of the outlier cases compared with 8% of non-outlier cases (corrected for disease location, p=6·8 × 10^−4^; appendix A). This finding suggests that we can indeed use genetics to identify small numbers of misclassified patients.

## Discussion

The successful identification of genetic variants associated with complex diseases such as inflammatory bowel disease has raised the exciting possibility of a more personalised approach to clinical management. In inflammatory bowel disease, this quest is particularly urgent because of the substantial heterogeneity in disease course, and individual response to therapy.^39^ Past studies in inflammatory bowel disease have established a genetic component of disease subphenotype,^14^, ^16^, ^18^, ^40^, ^41^ but these studies have been limited to a handful of candidate regions in modest numbers of patients. Our study, involving the universal application of standardised phenotyping by trained personnel on nearly 30 000 patients with inflammatory bowel disease from 49 centres worldwide, combined with matching genotypes from more than 150 000 variants, represents the definitive investigation to date into the genetic basis of subphenotypes of inflammatory bowel disease.

The only genome-wide significant associations we noted were between age of onset and disease location with variants at *NOD2*, MHC, and 3p21. Although the associations between ileal Crohn's disease and *NOD2*, and those between colonic Crohn's disease and the MHC, have been previously described,^14^, ^15^, ^16^, ^17^, ^18^, ^19^ our study has dissected the phenotype–location associations for the first time. Importantly, in Crohn's disease, we showed that *NOD2* is not associated with stricturing disease after accounting for disease location. These findings, and the rarity of long-term change in disease location compared with behaviour, suggest that location is a fundamental biological aspect of a patient's disease, whereas behaviour (like surgery or treatment history) is a marker of disease progression.

Our results for ulcerative colitis accord with the previously reported independent associations between the MHC and both extensive disease and colectomy.^17^, ^19^ Notably, the strongest associations with extensive ulcerative colitis are variants on the ancestral 8.1 *HLA* haplotype. This haplotype is a known recessive risk for primary sclerosing cholangitis,^42^, ^43^ a disease often associated with an extensive but quiescent form of ulcerative colitis.^44^ Although variants associated with susceptibility to Crohn's disease and those associated with susceptibility to ulcerative colitis are both predictive of disease location, the ulcerative colitis-associated variants are the most predictive. The variants associated with disease susceptibility are also slightly predictive of age at diagnosis.

Composite genetic risk scores from all 163 independent susceptibility signals were strongly associated with all our main subphenotypes, and these findings remained significant after excluding *NOD2* and MHC. This result accords with a similar finding for genetic risk scores in bipolar disorder,^45^ and hints at the possibility that such approaches might be broadly applicable for studying clinical heterogeneity of common diseases. This finding suggests that many or most risk variants for inflammatory bowel disease do contribute weakly to subphenotype. The relative dearth of individual single-nucleotide polymorphism associations with subphenotypes in our study, by contrast with those reported in similarly powered studies of inflammatory bowel disease susceptibility, suggests that the genetic variants studied here have a small effect, and that environmental factors (such as diet, microbiota, and smoking) might be strong contributors to the subphenotypes. However, an intriguing possibility, supported by the notable absence of any functional or pathway enrichment in the components of the genetic risk scores, is that current phenotypic classifications do not correspond strongly to underlying molecular entities. Of particular note is the genetic distinction seen between ileal Crohn's disease, colonic Crohn's disease, and ulcerative colitis. These disease types were identified as equally distinct entities on a genetic continuum: on multiple risk scores, colonic Crohn's disease was genetically intermediate between ileal Crohn's disease and ulcerative colitis, a finding that remained significant after excluding *NOD2*. This result substantiates the view that colonic versus ileal disease, rather than disease extent, is the primary clinical unit of Crohn's disease classification, and is further supported by the finding that both the genetic risk score and clinical complication rate in patients with ileocolonic disease is intermediate between that of patients with ileal disease and colonic disease. It will be of great interest, and potential clinical use, to see if application of these risk scores helps to classify the 10% of patients who are currently designated colonic inflammatory bowel disease unclassified.

The composite scores were also able to identify small numbers of patients with outlier scores who were much more likely to be misdiagnosed than a typical patient. These findings support the possible clinical usefulness of composite scores of multiple genetic variants, each of small effect. For example, genetic outliers could be excluded or specifically targeted for clinical trials to select more homogeneous groups. If these data were readily available, they might affect clinical decisions or inform risk–benefit discussions with patients. For example, the type of surgery offered to patients with refractory colitis crucially depends on whether they have ulcerative colitis or colonic Crohn's disease; poor outcomes (including pelvic sepsis, incontinence, and sexual dysfunction) of ileal pouch–anal anastomosis reconstruction surgery are much more common in patients with colonic Crohn's disease. Use of genetic risk scores to identify possible misdiagnoses in this group of patients could help to reduce this problem. Our data also suggest that genetic risk scores could augment biomarkers, such as faecal calprotectin, currently used for patient stratification in inflammatory bowel disease.

A limitation of our study is that the genetic variants tested were restricted to those present (and that passed quality control) on the Immunochip platform, designed for replication and fine mapping of potential immune-mediated disease loci. Therefore, there still might be important loci that determine disease behaviour, location, and age at onset but are independent of those that confer risk for inflammatory bowel disease (or other immune-mediated diseases), and had limited or absent coverage on the Immunochip. A high-powered GWAS, designed to assess possibly overlooked genetic determinants for these outcomes of phenotype expressivity, which uses our large collection of cooperatively phenotyped cases of inflammatory bowel disease with genomic DNA (and the pre-existing Immunochip genotypes), will likely be of great value.^46^

In summary, our research represents the largest genotype–phenotype study in inflammatory bowel disease done so far. Associations achieving genome-wide significance were identified at only three loci, suggesting that new clinical phenotypic classifications might need to be explored for inflammatory bowel disease, and the relation examined between subphenotypes and other omic profiles and environmental factors, including the microbiota.^47^ However, our data suggest that on the basis of genetic factors, inflammatory bowel disease is better classified into three distinct groups (ileal Crohn's disease, colonic Crohn's disease, and ulcerative colitis), and we would recommend that clinicians adopt this nomenclature in regular practice. We also show that, although genetic risk scores do not yet have widespread clinical use, they are already valuable in some contexts in inflammatory bowel disease, and their study in other complex disease phenotypes is warranted.

## Figures and Tables

**Table 1:** Phenotype distribution of primary cohort

		**Crohn's disease (n=16 902)**	**Ulcerative colitis (n=12 597)**	Inflammatory bowel disease*(n=29 838)
**Demographics**				
Sex				
	Male	7227 (44%)	6339 (51%)	13 738 (47%)
	Female	9257 (56%)	6027 (49%)	15 448 (53%)
	Missing	418 (3%)	231 (2%)	652 (2%)
Age at diagnosis (years)				
	Median (quartiles)	25 (19–36)	31 (22–24)	28 (20–40)
	<17 (A1)	2568 (18%)	1233 (11%)	3903 (15%)
	17–40 (A2)	9166 (64%)	6594 (58%)	15 854 (61%)
	>40 (A3)	2626 (18%)	3469 (31%)	6141 (24%)
	Missing	2542 (15%)	1301 (10%)	3940 (13%)
Family history				
	Yes	3471 (27%)	2232 (21%)	5778 (24%)
	No	9575 (73%)	8260 (79%)	18 005 (76%)
	Missing	3856 (23%)	2105 (17%)	6055 (20%)
Smoking status				21 718
	Smoker	3319 (28%)	1162 (12%)	4512 (21%)
	Ex-smoker	1665 (14%)	2739 (28%)	4436 (20%)
	Non-smoker	6752 (58%)	5853 (60%)	12 770 (59%)
	Missing	5166 (31%)	2843 (23%)	8120 (27%)
**Phenotypes**				
Disease location†				
	Ileal (L1)	3878 (31%)	..	..
	Colorectal (L2)	2933 (24%)	..	..
	Ileocolonic (L3)	5520 (44%)	..	..
	Other	154 (1%)	..	..
	Upper GI (L4)	1695 (14%)	..	..
	Missing	2777 (18%)	..	..
Disease extent†				
	Proctitis (E1)	..	1271 (12%)	..
	Left-sided (E2)	..	4087 (38%)	..
	Extensive (E3)	..	5212 (48%)	..
	Other	..	205 (2%)	..
	Missing	..	1822 (14%)	..
Disease behaviour†				
	Inflammatory (B1)	6196 (50%)	..	..
	Stricturing (B2)	3250 (26%)	..	..
	Penetrating (B3)	3054 (24%)	..	..
	Missing	2762 (18%)	..	..
Surgery‡				
	Yes	7257 (52%)	1932 (18%)	..
	No	6605 (48%)	8575 (82%)	..
	Missing	3040 (18%)	2090 (17%)	..

GI=gastrointestinal.

* Includes 255 patients with indeterminate colitis and 84 patients with missing exact diagnosis.

† Excludes data obtained with patient questionnaires (2658 patients).

‡ Surgery in ulcerative colitis refers to colectomy. Denominators for data are: 12 485 for disease location (L1, L2, L3, and other); and 11 717 for upper GI (L4) over non-missing information; and 12 485 for disease behaviour over non-missing B1, B2, and B3.

**Table 2:** Associations between genotype and age at diagnosis achieving genome-wide significance

	**MAF**	**Age at diagnosis of IBD**		**Age at diagnosis of Crohn's disease**		**Age at diagnosis of ulcerative colitis**	
		p value	β (SE)	p value	β (SE)	p value	β (SE)
**3p21 (*MST*)**							
rs35261698	0·306	6·34 × 10^−12^*	−0·06 (0·01)*	3·65 × 10^−07^	−0·06 (0·01)	3·90 × 10^−06^	−0·06 (0·01)
rs2172252	0·288	1·35 × 10^−12^*†	−0·06 (0·01)*†	2·93 × 10^−08^*†	−0·07 (0·01)*†	9·51 × 10^−06^	−0·06 (0·01)
rs3197999	0·281	2·73 × 10^−12^*	−0·06 (0·01)*	2·37 × 10^−08^*	−0·07 (0·01)*	2·18 × 10^−05^	−0·06 (0·01)
**6p21 (MHC)**							
rs3115674	0·116	3·42 × 10^−02^	−0·03 (0·01)	..	..	3·35 × 10^−02^	−0·04 (0·02)
rs4151651	0·034	..	..	..	..	1·15 × 10^−02^	−0·07 (0·03)
rs3129891	0·209	1·15 × 10^−06^	−0·05 (0·01)	..	..	1·43 × 10^−08^*†	−0·09 (0·02)*†
rs9268832	0·393	7·42 × 10^−09^*†	−0·05 (0·01)*†	4·56 × 10^−07^†	−0·06 (0·01)†	2·19 × 10^−03^	−0·04 (0·01)
rs482044	0·401	..	..	1·51 × 10^−02^	0·03 (0·01)	..	..
**16q12 (*NOD2*)**							
rs2066844 (p.R702W)	0·045	3·58 × 10^−07^	−0·08 (0·02)	1·21 × 10^−07^	−0·1 (0·02)	..	..
rs2066845 (p.G908R)	0·016	2·10 × 10^−04^	−0·1 (0·03)	5·50 × 10^−03^	−0·09 (0·03)	8·41 × 10^−03^	−0·15 (0·06)
rs2066847 (p.L1007fsX)	0·024	6·64 × 10^−16^*†	−0·16 (0·02)*†	2·04 × 10^−16^*†	−0·17 (0·02)*†	..	..

Loci are listed by single nucleotide polymorphism. Age at diagnosis assessed by linear regression analysis on normalised data for Crohn's disease and ulcerative colitis; IBD assessed by meta-analysis of Crohn's disease and ulcerative colitis data. Effect size is given as standard deviation unit (standard error of effect). MAF=minor allele frequency. IBD=inflammatory bowel disease. ..=non-significant associations (p_nominal_<0·05).

* Genome-wide significant associations.

† The most significant association pews per subphenotype, if genome-wide significant.

**Table 3:** Associations between genotype and disease location, behaviour, extent, surgery, and colectomy achieving genome-wide significance

	**MAF**	**Crohn's disease**							**Ulcerative colitis**			
		**Disease location**			**Disease behaviour**		**Surgery**		**Disease extent**		**Colectomy**	
		p value	OR (95% CI), ileocolonic *vs* colonic	OR (95% CI), ileal *vs* colonic	p value	OR (95%CI)	p value	HR (95% CI)	p value	OR (95% CI)	p value	HR (95% CI)
**3p21 (*MST1*)**												
rs2172252	0·288	3·10 × 10^−02^	1·07 (1·00–1·13)	1·10 (1·02–1·19)	..	..	..	..	..	..	..	..
rs3197999	0·281	2·10 × 10^−02^	1·08 (1·02–1·15)	1·10 (1·02–1·19)	..	..	..	..	..	..	..	..
**6p21 (MHC)**												
rs3115674	0·116	3·00 × 10^−03^	0·88 (0·80–0·97)	0·81 (0·72–0·91)	4·00 × 10^−03^	0·89 (0·82–0·96)	..	..	5·22 × 10^−15^*†	1·43 (1·30–1·58)*†	..	..
rs4151651	0·034	2·42 × 10^−10^*	0·71 (0·62–0·81)*	0·58 (0·50–0·68)*	2·50 × 10^−02^	0·87 (0·77–0·98)	..	..	..	..	6·05 × 10^−12^*†	1·72 (1·47–2·00)*†
rs6930777	0·112	8·13 × 10^−23^*†	0·68 (0·62–0·75)*†	0·58 (0·52–0·65)*†	2·00 × 10^−03^	0·89 (0·82–0·96)	..	..	..	..	2·49 × 10^−07^	1·36 (1·21–1·52)
rs3129891	0·209	..	..	..	8·00 × 10^−03^	0·92 (0·87–0·98)	..	..	3·22 × 10^−10^*	1·24 (1·17–1·32)*	..	..
rs9268832	0·393	1·40 × 10^−02^	1·03 (0·97–1·09)	0·94 (0·87–1·02)	4·00 × 10^−03^	0·93 (0·89–0·97)	1·45 × 10–02	0·95 (0·90–0·99)	6·59 × 10^−05^	1·12 (1·06–1·19)	..	..
rs482044	0·401	2·38 × 10^−09^*	1·15 (1·08–1·22)*	1·25 (1·16–1·35)*	8·46 × 10^−06^	1·11 (1·07–1·15)	1·84 × 10–02	1·05 (1·01–1·10)	1·57 × 10^−05^	0·88 (0·83–0·93)	2·19 × 10^−07^	0·79 (0·73–0·87)
rs77005575	0·439	1·00 × 10^−15^*	1·23 (1·16–1·30)*	1·33 (1·23–1·44)*	2·82 × 10^−10^*†	1·16 (1·12–1·21)*†	9·20 × 10–04	1·08 (1·03–1·12)	3·24 × 10^−03^	0·92 (0·87–0·98)	1·55 × 10^−04^	0·85 (0·78–0·93)
**16q12 (*NOD2*)**												
rs2066844 (p.R702W)	0·045	2·50 × 10^−26^*	1·61 (1·43–1·81)*	1·94 (1·72–2·18)*	1·76 × 10^−06^	1·21 (1·12–1·31)	4·67 × 10–03	1·10 (1·03–1·18)	..	..	..	..
rs2066845 (p.G908R)	0·016	2·77 × 10^−09^*	1·59 (1·31–1·93)*	1·82 (1·50–2·21)*	7·17 × 10^−05^	1·28 (1·14–1·44)	2·87 × 10–03	1·17 (1·06–1·30)	..	..	..	..
rs2066847 (p.L1007fsX)	0·024	1·01 × 10^−35^*†	1·89 (1·62–2·21)*†	2·50 (2·14–2·92)*†	5·73 × 10^−10^*†	1·31 (1·21–1·42)*†	2·04 × 10–13*†	1·31 (1·22–1·40)*†	..	..	3·55 × 10^−02^	1·32 (1·02–1·70)

Loci are listed by single nucleotide polymorphism. Disease location assessed by multinomial logistic regression analysis; disease behaviour by ordinal logistic regression analysis (effect size is odds ratio [95% CI] for B2 versus B1, which is also equivalent to B3 vs B2+B1); and disease extent by binomial logistic analysis. Surgery and colectomy assessed by survival analysis under a Weibull distribution. MAF=minor allele frequency. OR=odds ratio. HR=hazard ratio. ..=non-significant associations (p_nominal_<0·05).

* Genome-wide significant associations.

† The most significant association per locus per subphenotype, if genome-wide significant.

**Table 4:** Variance explained by demographic and genetic factors for disease location in adult onset of Crohn's disease

	**Beta**	**SE**	**p value**	**R^2^**
Ever smoker	−0·041	0·108	7·00 × 10^−1^	0·01%
Smoker at diagnosis	0·473	0·117	5·34 × 10^−5^	1·53%
Age at diagnosis	−0·033	0·005	1·50 × 10^−9^	2·14%
Year of birth	−0·010	0·005	6·94 × 10^−2^	0·19%
rs6930777 (MHC)	−0·302	0·082	2·19 × 10^−4^	0·54%
rs77005575 (MHC)	0·190	0·055	5·21 × 10^−4^	0·55%
NOD2*	0·532	0·070	2·60 × 10^−14^	3·23%
Genetic risk score†	0·165	0·038	1·61 × 10^−5^	1·01%
Genetic parameters	..	..	..	5·5%
Genetics and smoking	..	..	..	6·8%
All parameters‡	..	..	..	8·03%

* Number of risk alleles at the three *NOD2* hits.

† Crohn's disease versus ulcerative colitis (CD *vs* UC) genetic risk score (without *NOD2* and MHC).

‡ The total R^2^ for these parameters, excluding the principal component used to account for population stratification. In view of the correlation structure, this is not expected to be equivalent to the sum of R^2^ obtained for each parameter.

**Table 5:** Variance explained by demographic and genetic factors for disease extent in adult onset of ulcerative colitis

	**Beta**	**SE**	**p value**	**R^2^**
Ever smoking	0·1268	0·0856	1·38 × 10^−1^	0·12%
Current smoking	0·1229	0·1179	2·97 × 10^−1^	0·06%
Age at diagnosis	−0·0234	0·0055	2·41 × 10^−5^	1·10%
Year of birth	0·0008	0·0055	8·79 × 10^−1^	0·00%
rs3115674	0·3795	0·0832	5·08 × 10^−6^	0·80%
Genetic risk score*	0·0784	0·0502	1·18 × 10^−1^	0·09%
Genetic parameters	..	..	..	0·9%
Genetics and smoking	..	..	..	1·1%
All parameters†	..	..	..	2·39%

* Crohn's disease versus ulcerative colitis (CD *vs* UC) genetic risk score (without *NOD2* and MHC).

† The total R^2^ for these parameters, excluding the principal component used to account for population stratification. In view of the correlation structure, this is not expected to be equivalent to the sum of R^2^ obtained for each parameter.
