# Supplementary material for: Inherited determinants of Crohn's disease and ulcerative colitis phenotypes: a genetic association study
Source: Lancet. 2016 Jan 9;387(10014):156–67. doi: 10.1016/S0140-6736(15)00465-1 (PMC4714968; doi:10.1016/S0140-6736(15)00465-1)
Supplement: Supplementary appendix [file mmc1.pdf]

THE LANCET

## Supplementary appendix

This appendix formed part of the original submission and has been peer reviewed. We post it as supplied by the authors.

Supplement to: Cleynen I, Boucher G, Jostins L, et al. Inherited determinants of Crohn's disease and ulcerative colitis phenotypes: a genetic association study. *Lancet* 2015; published online Oct 19. http://dx.doi.org/10.1016/S0140-6736(15)00465-1.

**Colonic Crohn’s disease is genetically distinct from ileal Crohn’s disease and ulcerative colitis**

Cleynen I*, Boucher G*, Jostins L* et al

# Appendix

**Overview of content**

1. Supplementary Methods, p3-7
    1. Classification of phenotypes (p3)
    2. Data collection, curation and QC (p3)
    3. Survival analyses (p3)
    4. Genotype calling and QC (p4)
    5. Age-of-onset, surgery and upper GI association (p4)
    6. Model selection (classifying phenotypes using risk variants) (p5)
    7. Multinomial and ordinal data analysis (Trinculo) (p8)
    8. Calculation of genetic risk scores and cross-validation of predictive model (p8)
2. Supplementary Tables, p9-12
    1. Supplementary Table 1: Description of recruiting centres (Appendix B)
    2. Supplementary Table 2: Model selection and cross-validation (page 10)
    3. Supplementary Table 3: Phenotype distribution of replication cohort (page 11)
    4. Supplementary Table 4: Suggestive ($p<1x10^{-05}$) genetic association results for all studied phenotypes (Appendix B)
    5. Supplementary Table 5: Genotype-phenotype association results for the 163 known IBD loci (Appendix B)
    6. Supplementary Table 6: MHC associations for disease susceptibility and disease sub-phenotypes (Appendix B)
    7. Supplementary Table 7: Predictive accuracy of risk scores based on SNPs and HLA types, assessed by cross-validation (p13)
    8. Supplementary Table 8: Clinical characteristics for CD patients with low and high CDvsUC risk scores (p14)
    9. Supplementary Table 9: Review of diagnosis and genetic risk score (p15)
3. Supplementary Figures, p16-34
    1. Supplementary Figure 1: CD disease location versus age at diagnosis (p16)
    2. Supplementary Figure 2: UC disease extent versus age at diagnosis (p17)
    3. Supplementary Figure 3: CD disease behaviour versus age at diagnosis (p18)
    4. Supplementary Figure 4: Survival analysis for time to first complication after CD diagnosis (p19)
    5. Supplementary Figure 5: Survival analysis for time to first surgery after CD diagnosis (p20)
    6. Supplementary Figure 6: Survival analysis for time to colectomy after UC diagnosis (p21)

7. Supplementary Figure 7: Time to first complication (B2 or B3) after CD diagnosis per disease location (p22)
8. Supplementary Figure 8: MHC region plot for CD age at diagnosis (p23)
9. Supplementary Figure 9: MHC region plot for UC age at diagnosis (p24)
10. Supplementary Figure 10: MHC region plot for CD location (p25)
11. Supplementary Figure 11: MHC region plot for CD behaviour (p26)
12. Supplementary Figure 12: MHC region plot for UC extent (p27)
13. Supplementary Figure 13: MHC region plot for CD surgery (p28)
14. Supplementary Figure 14: MHC region plot for UC colectomy (p29)
15. Supplementary Figure 15: MHC association for CD disease location versus UC susceptibility (p30)
16. Supplementary Figure 16: MHC association for CD disease location versus CD susceptibility (p31)
17. Supplementary Figure 17: Effect sizes of genetic risk scores (GRS) for disease location, disease behaviour and age at diagnosis excluding genome-wide significant loci (NOD2, MHC and MST1) (p32)
18. Supplementary Figure 18: Consistent positioning of purely colonic CD as intermediate between purely ileal CD and UC on four different risk scores (p33)
19. Supplementary Figure 19: BIC-based phenotype model selection for UC, purely colonic CD (CCD) and purely ileal CD (ICD) (p34)

4. References (p35)
5. International IBD Genetics Consortium list of participants and affiliation (p36)

## Supplementary Methods

### Classification of phenotypes

*Crohn's Disease Phenotypes*

Crohn's disease cases were classified by disease location, disease behaviour, and age at diagnosis. Disease location was characterized according to The Montreal Classification (L1- disease location limited to ileum; L2- disease location limited to colon; L3 – disease located in ileum and colon; L4 – isolated upper gastrointestinal disease), as was disease behaviour (B1 – non-stricturing, non-penetrating; B2- stricturing disease; B3 – internal penetrating disease. Subjects were also characterized as to whether they had perianal disease or not. Surgery was defined as abdominal surgery for complication or treatment. Age at onset was reported as a continuous trait, and according to the Montreal Classification (A1 – age of onset 16 years or younger; A2 – age of onset from 17 to 40 years; and A3 – age of onset greater than 40 years of age). Smoking was defined as "ever smoker" or "never smoker" at the time of diagnosis of CD. Family history of IBD was designated as positive if the proband had any first or second degree relatives with IBD.

*Ulcerative colitis phenotypes*

The Montreal Classification was also used to characterize the ulcerative colitis clinical phenotypes of disease extent and age of onset. Disease extent was defined as: E1 - ulcerative proctitis (limited to the rectum); E2 – left sided or distal disease (distal to the splenic flexure); and E3 – extensive disease (extending proximal to the splenic flexure). Surgery was defined as colectomy for complication or treatment. Age at onset, smoking status and family history were defined similarly as for Crohn's disease (see above).

### Data collection, curation and QC

Phenotype data were solicited directly from the recruiting physicians using a detailed instruction manual describing each data item and the constraints it must meet to be considered valid. Data were submitted to the NIDDK IBD Genetics Consortium Data Coordinating Center, where they were programmatically checked against a set of validation rules. These included both individual range checks for each item and tests for consistency among items, the latter including: checking disease location for consistency with diagnosis; verifying that all dates were consistent within a subject; verifying that the set of items describing surgical history or smoking history were internally consistent; and checking that treatment variables and extra-intestinal manifestations were consistent with affection status. All validation errors were communicated back to the data submitter, and were resolved through resubmission of the affected records. In cases where an obvious error or inconsistency could not be resolved, the corresponding data were excluded from analysis (no imputation or guessing was performed centrally). The resulting data were then compiled into a single dataset for analysis.

A second round of quality control was then applied to the compiled dataset. Within- and between-center information and referral to the analytic models was used to identify possible further encoding issues, discrepancies in phenotype definitions or bias in sampling. External information such as official smoking rates and assessments by clinicians was used to verify plausibility of the data and representativeness of the sample. We investigated for non-random missing values, which could affect the conclusions. For example, we compared the phenotype distribution of centers with more missing values to those with lower missing rates and, when other phenotypes were available, we compared the distribution of these other phenotypes vs the missing values. We identified a few issues that were due to encoding issues or the use of patient questionnaires. These have been addressed by correcting the data or removing the center.

### Survival analyses

For disease behaviour in CD, year of diagnosis and year of last review were used to calculate disease duration and define intervals for the event. For a patient with known complicated disease at last review, the complication event happened before that time. For an individual without complicated disease at last review, the event could only happen after that time. In this context, every interval is left or right censored, which is an extreme situation known as "case-one" interval. The survival analysis based on such censored data relies on the hypothesis that the censoring is independent from the event. In the context of this study, it is likely that time at last review is mostly, but not completely, independent from the event, i.e., more complicated cases may need closer follow-up. For time to surgery, we proceeded in a similar manner, except for patients with information about year of surgery, which allowed us to reduce the interval to a 1 year window.

Kaplan-Meier curves were derived from these interval data. The conditional analyses were performed based on disease location at time of last review. Given the fact that disease location can change slightly with time, the interpretation of these curves should be done accordingly. In this context, the curves estimate the proportion of patients who underwent surgery or had complicated disease sometime after diagnosis, conditional on disease location at that time.

Smoothed distributions of phenotypes versus age at diagnosis and disease duration were estimated based on the average of a 2-year and a 4-year window centered at each time point. Confidence intervals at each time point were estimated based on the ordinary bootstrap standard error estimate. To be noted, these estimates of proportions are not corrected for other parameters. In particular, distribution of phenotype versus age at diagnosis is not corrected for disease duration, which is expected to be longer for younger patients. For disease behaviour, the estimates were similar to those obtained by survival analysis.

**Genotype calling and QC**

Initial genotype calling was done per genotyping batch (n=35) using Illumina'sBeadStudio. Before centrally re-calling all data using optiCall v.0.6.2,[1] all samples with outlying autosomal intensity (>5 sds in batch) were removed from further analyses, as well as all samples with > 5% missing data at SNPs with < 10% missing data within batches. Samples were classified into continental groups using principal component analysis (PCA) using a HapMap3 reference set, and into males and females using X and Y intensities. Related and duplicated samples (pi_hat > 0.2), samples whose X and Y intensities are inconsistent with stated sex and samples whose PCA results are inconsistent with stated race were flagged within batches. More specifically, we carried out principal component analysis on the HapMap phase 3 samples, using only those variants that were present in both HapMap3 and the Immunochip. We fitted a four-cluster multivariate Gaussian mixture model that grouped the HapMap samples into four ethnic groups (European, African, East Asian and South Asian) using the first two principal components. Each Immunochip sample was then projected onto these same two principal component axes, and was assigned to one of the four ethnic groups according to their relative likelihoods (equivalent to a Gaussian mixture model classification with a uniform prior). Samples that had a posterior probability less than 50% for their stated ethnicity, or were not within 8 standard deviations of any population, were flagged as inconclusive. For this paper we only analyzed samples that were classified conclusively as European. OptiCall clustering was then performed for each batch separately, with a Hardy-Weinberg Equilibrium (HWE)-threshold of $1\times10^{-15}$, HWE blanking disabled and a genotype call threshold of 0.7. HWE was calculated conditional on predicted ethnicity, and related individuals were removed from this calculation. Sex chromosomes were called using the predicted sex.

After re-calling, all variants failing the HWE test, or with different missing genotype rates (using a chi-square test) in affected and unaffected individuals, or with significantly different allele frequencies across the batches based on a false-discovery rate (FDR) threshold of $1\times10^{-5}$ for each test were removed. In addition, variants with missing genotype rate > 2% across the entire collection, or > 10% in a single batch were removed. Variants that only failed one QC criteria in a single batch (i.e. would pass the QC if this particular batch was ignored) were set to missing in the failed batch, otherwise they were removed from the entire dataset. Individuals were removed if they showed a missing genotype rate >2%, had a significantly higher or lower inbreeding coefficient (F) (calculated using the '--het' option in PLINK[2]) at FDR <0.01, and showed a high level of relatedness (PI_HAT ≥ 0.4) calculated on IBS distance between all individuals. Calculation of inbreeding coefficient and sample relations were calculated on a LD pruned dataset to keep only independent variants.

In order to control for population stratification while avoiding the possible bias introduced by the enrichment of associated alleles in the dataset, principal components (PCs) were computed based on the control samples, and then applied to the affected samples. PCs were computed based on a set of 18,123 independent (LD pruned) SNPs across the Immunochip. To generate the LD pruned SNPs, we removed variants in long range LD[3] and pruned the common variants (MAF>0.05) three times using the '--indep' option in PLINK (with window size of 50, step size of 5 and VIF threshold of 1.25). The genomic inflation factor for disease susceptibility ($\lambda$) was estimated from a set of 3,120 "null" SNPs (chosen based on GWAS of schizophrenia, psychosis and reading/mathematics ability), using different subsets of PCs. Based on these and investigation of contribution of individual SNPs to the components (loadings), we selected to use the first five PCs to control for population stratification in all the analyses.

The cluster plots of all SNPs mentioned in the text or figures of the manuscript were manually inspected by three independent assessors, using the program Evoker v2.2.[4] SNPs were marked as poorly genotyped if they did not show three clear clouds in every batch. No SNPs failed this inspection.

Variants in the MHC, including SNPs, HLA alleles at *HLA-A*, *HLA-C, HLA-B*, *HLA-DRB1*, *HLA-DQA1*, *HLA-DQB1*, *HLA-DPA1 and HLA-DPB1* were imputed using SNP2HLA imputation pipeline.[5] Only variants with more than 0.5% frequency were tested for association. Imputed data and primary results for CD and UC were obtained from the fine mapping project in the MHC.[6]

### Age-of-onset, surgery and upper GI association

To test for association between age-of-onset and genotypes, we quantile-normalized the CD and UC age-of-onset data separately, carried out separate linear regression analysis, and combined the results using a fixed-effect inverse variance weighted meta-analysis. Binary logistic regression was used to analyze upper gastrointestinal involvement and perianal disease (irrespective of other disease behaviors or location). To measure association with time to first surgery in CD and time to colectomy in UC, a parametric survival regression using a Weibull distribution was fitted using the R package "survival". The point estimates and confidence intervals on the hazard ratio were computed from the parameter estimates as follows: let $\gamma$ be the effect size estimate and $s$ be the estimate of the (log) scale parameter ($s$=log($\sigma$)). Given the parametrization used, the hazard ratio estimate is $e^{\beta}$, where $\beta = -\frac{\gamma}{e^s}$. The standard error estimate for $\beta$ was approximated using the delta method:[7]

$$\text{var}(\beta) \approx \frac{1}{e^{2s}}\left(\text{var}(\gamma) + \gamma^2\text{var}(s) - 2\gamma\text{cov}(\gamma, s)\right)$$

where var(γ) and var(s), are the variance estimates of $\gamma$ and $s$, respectively, and cov(γ,s) is the covariance estimate obtained from the covariance matrix of the model. The 95% confidence estimates were computed under the hypothesis of a Gaussian distribution for $\mu$.

### Replication

The replication cohort was obtained from the IIBDGC repository from patients with available phenotype information and genome-wide imputed data.[8] Patients included in the discovery phase of this study were discarded. A large subset of patients were included from cohorts of pediatric cases of IBD. Given the difference in phenotypes and age of these pediatric cases, these patients were analyzed as a single cohort. The remaining samples had phenotype distribution similar to the discovery cohort and were analyzed together. Results from these two analyses were combined by meta-analysis. A limitation of this replication is the lower variance of phenotypes in the pediatric cohort, in particular for age at diagnosis. For this reason, the pediatric cohort was not used for the age of onset analysis. The pediatric cohort was also not used for the analysis of time to surgery, because the data was unavailable (**supplementary table 3**).

### Model selection (Classifying phenotypes using risk variants)

<u>Aim of the method</u>

As well as containing information about the relationship between genotype and phenotype, risk variants can also contain information about the relationship between different phenotypes. For instance, NOD2 risk variants are most common in ileal CD patients, rarest in colonic CD patients, and of intermediate frequency in ileocolonic patients. This suggests that ileocolonic CD patients are in some sense intermediate between ileal and colonic CD patients (as is also supported by clinical evidence).

The method outlined below provides a systematic framework for carrying out such inferences. It combines information across multiple predictors (both risk variants and risk scores) to test hypotheses about genetic relationships between different phenotypes. We fit a number of genotype-phenotype models by maximum likelihood, and compare them using model selection carried out with the Bayesian Information Criterion. We additionally use cross-validation to validate the results of our method.

The information produced by this method is useful for two reasons. Firstly, it provides potential biological and clinical insight into the phenotypes under study. Secondly, it suggests a natural statistical framework for carrying out a genotype-phenotype scan: once an optimal model for the relationship between a genotype and multiple phenotypes has been established, this model can be used to carry out regression and likelihood ratio test. The genome-wide scans described in the main text were all carried out using the model optimally selected for each phenotype using this method.

<u>Description of models and model selection</u>

*Overview of logistic models*

We fitted various constrained versions of two forms of logistic regression model to the data: multinomial and ordinal logistic models (both described in Chapter 8 of [9]). Constrained versions were constructed by fixing certain parameters to zero, or fixing pairs of parameters to be equal to each other, as described below. All models had three phenotype categories; while they all generalize to arbitrary numbers of phenotypes, the number of constrained versions increases exponentially with the number of phenotypes.

For individual $i \in \{1, \ldots, N\}$ we denote the observed phenotype as $y_i \in \{1, \ldots, K\}$, where $K$ is the number of possible outcomes; and predictor variables (genotypes and risk scores) as $\boldsymbol{x_i} \in \mathbb{R}^{L+1}$, with $L$ the number of predictors ($x_{i0} = 1$, for an intercept). We will denote as $\boldsymbol{\beta_j}$ the vector of regression parameters for outcome *j* and by $\beta = \{\beta_{js}\}_{j \in \{1,\ldots,K\}; s \in \{0,\ldots,L\}}$ the complete set of regression parameters in the model. We will denote the probability of an outcome $Y = j$, conditional on the predictors as:

$$p_j(x) = P(Y = j | \boldsymbol{x}, \beta),$$

from which we can generate the general form of the likelihood for model *M*:

$$L(M) = \prod_{i=1}^{N} p_{y_i}(\boldsymbol{x_i}).$$

*Multinomial logistic models*

The multinomial model is a simple generalization of the logistic model to multiple independent and mutually exclusive categories. The probability of an outcome *j* is equal to:

$$p_j(\boldsymbol{x}) = \frac{\exp(\boldsymbol{\beta}_j^T \boldsymbol{x})}{\sum_{s=1}^{K} \exp(\boldsymbol{\beta}_s^T \boldsymbol{x})}.$$

We set the first category as an arbitrary reference, with $\boldsymbol{\beta}_1 = \vec{0}$.

For the constrained multinomial (or pseudobinomial) model for three phenotypes, we set one of the phenotypes to the reference category, and for the other two phenotypes we set $\beta_{2s} = \beta_{3s}$ for all predictors other than the intercepts, i.e. for $s \in \{1, \ldots, L\}$. This gives three models, corresponding to three different reference phenotypes. For each model the two non-reference phenotypes are considered identical to each other for the effect of the predictors, and different from the reference phenotype.

*Ordinal logistic models*

Instead of a set of independent categories, the ordinal logistic with cumulative link model treats phenotypes as a series of ordered, embedded classes. This means that class 2 contains phenotypes 1 and 2, class 3 contains phenotypes 1, 2 and 3 and so on. This model therefore represents a phenotype that has a natural ordering.

The ordinal model with cumulative link is best represented as the probability of being in a given class:

$$P(Y \leq j | \boldsymbol{x}, \beta) = \begin{cases} \dfrac{1}{1 + \exp\left(-\boldsymbol{\beta}_j^T \boldsymbol{x}\right)}, & \text{for } j \in \{1, \ldots, K-1\} \\ 1, & \text{for } j = K. \end{cases}$$

In this model, there are no parameters corresponding to the last category, i.e. $\boldsymbol{\beta}_K$ is not defined.

Thus the probability of an individual having any given phenotype, conditional on the predictors is:

$$p_j(\boldsymbol{x}) = P(y_i \le j\,|\boldsymbol{x},\beta) - P(y_i \le j-1\,|\boldsymbol{x},\beta)$$
$$= \begin{cases} \dfrac{1}{1+\exp\left(-\boldsymbol{\beta}_j^T\boldsymbol{x}\right)}, & \text{for } j = 1 \\ \dfrac{1}{1+\exp\left(-\boldsymbol{\beta}_j^T\boldsymbol{x}\right)} - \dfrac{1}{1+\exp\left(-\boldsymbol{\beta}_{j-1}^T\boldsymbol{x}\right)}, & \text{for } j \in \{2,\dots,K-1\} \\ 1 - \dfrac{1}{1+\exp\left(-\boldsymbol{\beta}_{j-1}^T\boldsymbol{x}\right)}, & \text{for } j = K. \end{cases}$$

For the constrained ordinal model (or proportional odds model) for two phenotypes, we assign the three phenotypes to some order, and set $\beta_{1s} = \beta_{2s}$ for all predictors other than the intercepts, i.e. for $s \in \{1,\dots,L\}$. This gives us three different models corresponding to the three possible non-equivalent orderings (as models with ordering 1, 2, 3 are equivalent to models with ordering 3, 2, 1 with negative effect sizes). Each represents a model where phenotypes are simply ordered, with each predictor increasing the odds of being in a class by the same amount.

*Parameter estimation*

We estimate the parameters of the models, subject to certain constraints, by maximum likelihood. Such models are often fitted using an efficient Newton or Quasi-Newton method (as in the Trinculo analysis below). However, standard implementations are not open source, and so here we fit all models by simulated annealing, using the freely available R package GenSA [ http://journal.r-project.org/archive/2013-1/xiang-gubian-suomela-etal.pdf], This is less efficient, but easier to implement, as it does not require calculation of derivatives of the log likelihood. For the constrained ordinal and general multinomial we check the solutions against the Trinculo output (described below).

*Model comparison*

To compare models we fit the general unconstrained forms of both the multinomial and the logistic models, as well as three versions of each model with certain constraints on $\beta$ as described before. To perform model selection we use a penalized likelihood: the Bayesian Information Criterion (BIC)[10], which can compare alternative parameterizations and non-nested models. While the BIC is perhaps best known for its use in feature selection, in our case the features (predictors) are held constant and instead we are using BIC to select between probability models and parameter constraint. This is similar to how the BIC is used to pick the link function in a generalized regression analysis (e.g. section 5.5.2 of [11]). Under certain assumptions the calculated BICs produce model weights for each model that approximate the posterior probability that this model is the "best" model (informally, the model that minimizes the information loss compared to the true model, see Section 5.5.5 of [11]). For more on BIC weights, and when they can be properly interpreted as posteriors, see Section 6.4.3 of [12].

The ordinal and multinomial models handle covariates differently: covariates are included as further predictors, and are thus interpreted through differing probability models (either ordinal or multinomial). This could induce bias when handling principal components, as the selected model may be the one that most accurately models stratification, rather than the one that most accurately models the true genetic effects. As a result, we do not include PCs in the models directly: instead, we remove variation due to PCs 1-5 from the predictors themselves, by linearly regressing the predictors against the PCs and taking the residuals.

When classifying CD subphenotypes, we use the CDvsUC risk score (minus HLA and *NOD2* variants), plus the three *NOD2* variants and the HLA variant from Table 3. When classifying UC subphenotypes, we use the CDvsUC risk score and the HLA variant from Table 3. We also ran a large model for colonic CD, ileal CD and UC that included all 193 SNPs and all 23 imputed HLA types as separate predictors, in order to avoid over fitting from the risk scores.

Cross-validation

To ensure that the approach described above was working as expected, we used an alternative scheme (cross-validation) to test for differences in the predictive power of the different models.

We carry out 10-fold cross validation by splitting the dataset up into 10 equally sized non-overlapping test sets. For each test set we fitted each of the models on all individuals that were not in the test set, and used the parameters estimated to assign the estimated probabilities to each individual for each outcome in the test set. From these probabilities we then calculated the cross-validation likelihood using equations above to produce a

measure of the predictive power of each model that is free from overfitting. We also calculated the probability given to the correct phenotype for each individual during the cross-validation ($p_{y_i}(x_i)$), and used a non-parametric Wilcoxon signed-rank test for differences between pairs of model's predictions.

**Supplementary table 2** shows the results of both the BIC analysis and the cross validation for CD location, CD behavior and UC extent. In general, the BIC and the cross-validation gave very similar results. For CD location the BIC analysis and cross-validation produced exactly the same ordering of models, and the best model (the general model) had both a BIC weight of >99.9% and had a significantly higher likelihood than all other models under the non-parametric test. For CD behavior, the BIC analysis and the cross-validation supported the same best model (B1>B2>B3, with a BIC weight of 98%), though the non-parametric cross-validation test found that the B1>B2+B3 and general models were not significantly better than the best model. For UC extent, the BIC and cross-validation analyses gave almost identical orderings, with the only difference being that the cross-validation analysis slightly favored the general model, as opposed to the E1+E2>E3 model favored by the BIC analysis, and many models could not be distinguished under the non-parametric test.

### Multinomial and ordinal data analysis (Trinculo)

We developed custom software to efficiently fit certain multicategory logistic models, allowing multi-phenotype analyses to be run genome-wide. The multinomial and ordinal models described above are fitted using the Newton-Raphson method, and the statistical significances of the associations are tested using likelihood ratio tests. The C++ program (called Trinculo) takes in plink-formatted genotype, phenotype and covariate data, and is available from https://sourceforge.net/projects/trinculo/.

This software was used to perform the genome-wide scans for CD location and CD behavior shown in Table 2. The CD location scan was carried out under a general multinomial model, and the CD behavior scan was carried out under a proportional odds (constrained) ordinal logit model with ordering B1<B2<B3. The scan for UC extent was carried out under a binomial model using plink. These models were each selected as they were the optimal found during the model selection stage described above (see **Supplementary table 2** for details).

### Calculation of genetic risk scores (GRS) and cross-validation of predictive model

All risk scores initially included the 193 independent IBD associations.[8] To generate scores we first calculated association p-values and odds ratios for the phenotype of interest using a logistic regression model, conditional on the first 5 principal components. All SNPs with p<0.05 were included in the score, using the odds ratios estimated from the logistic model and allele frequencies taken from controls. Standard logistic risk scores were then computed for all samples using the R package "Mangrove" [http://cran.r-project.org/web/packages/Mangrove/index.html].[13] Similarly, risk scores were calculated using all SNPs excluding those in the *MHC*, and *NOD2*. Risk scores were generated for CD vs controls, UC vs controls, CD vs UC, ileal CD vs UC and colonic CD vs UC.

To assess the predictive accuracy of the risk scores in CD location (the most strongly predictable subphenotype), we split the data into a training set consisting of non-UK samples, and a validation set consisting of all UK samples. The models were fitted in the training set (with models trained to separate CD vs UC, or directly separate purely ileal and purely colonic CD), and classification accuracy to classify ileal vs colonic CD was assessed in the validation set using the area under the ROC curve (AUC). We also tested if inclusion of 23 IBD-associated imputed HLA alleles would improve these scores. Note that, other than this test, HLA alleles were not used in any other risk scores reported in this paper.

## Supplementary Tables

### Supplementary Table 1: Description of recruiting centres

See Appendix B

## Supplementary Table 2: Model selection and cross-validation

| | Bayesian information criterion analysis | | | Cross-validation analysis | | |
|---|---|---|---|---|---|---|
| **Phenotype model** | **BIC** | **Delta BIC** | **BIC weight** | **2L** | **delta -2L** | **P-value*** |
| | | | **CD location** | | | |
| L1>L2>L3 | -28544.24 | -480.82 | 3.9018E-105 | -28560.12 | -521.82 | 3.68E-81 |
| L2>L1>L3 | -28432.66 | -369.24 | 6.61533E-81 | -28445.22 | -406.92 | 1.60E-63 |
| L1>L3>L2 | -28103.04 | -39.62 | 2.49244E-09 | -28114.56 | -76.26 | 6.63E-06 |
| L1>L2+L3 | -28344.2 | -280.78 | 1.07003E-61 | -28357.64 | -319.34 | 5.39E-34 |
| L1+L2>L3 | -28553.66 | -490.24 | 3.5135E-107 | -28567.98 | -529.68 | 1.58E-73 |
| L1+L3>L2 | -28089.16 | -25.74 | 2.57412E-06 | -28100.8 | -62.5 | 6.13E-24 |
| **General** | **-28063.42** | **0** | **0.999997423** | **-28038.3** | **0** | **NA** |
| | | | **CD behaviour** | | | |
| **B1>B2>B3** | **-28429.6** | **0** | **0.98270684** | **-28441.18** | **0** | **NA** |
| B2>B1>B3 | -28630.9 | -201.3 | 1.90847E-44 | -28640.26 | -199.08 | 1.03E-54 |
| B1>B3>B2 | -28503.16 | -73.56 | 1.0449E-16 | -28514.98 | -73.8 | 3.37E-16 |
| B1>B2+B3 | -28437.68 | -8.08 | 0.017293157 | -28450.6 | -9.42 | 0.2404106 |
| B1+B2>B3 | -28520.76 | -91.16 | 1.575E-20 | -28530.48 | -89.3 | 6.20E-32 |
| B1+B3>B2 | -28630.58 | -200.98 | 2.23961E-44 | -28641.02 | -199.84 | 5.45E-50 |
| General | -28468.76 | -39.16 | 3.08275E-09 | -28442.02 | -0.84 | 0.1995 |
| | | | **UC extent** | | | |
| E1>E2>E3 | -20340.78 | -3.04 | 0.173292464 | -20347.7 | -4.9 | 9.35E-05 |
| E2>E1>E3 | -20344.06 | -6.32 | 0.03361528 | -20351.3 | -8.5 | 0.13 |
| E1>E3>E2 | -20393.46 | -55.72 | 6.30178E-13 | -20400.22 | -57.42 | 0.0009672 |
| E1>E2+E3 | -20398.06 | -60.32 | 6.31809E-14 | -20404.32 | -61.52 | 2.86E-11 |
| **E1+E2>E3** | **-20337.74** | **0** | **0.79233217** | **-20344.8** | **-2** | **3.38E-13** |
| E1+E3>E2 | -20366.18 | -28.44 | 5.28737E-07 | -20373.52 | -30.72 | 0.05911 |
| General | -20351.64 | -13.9 | 0.000759557 | -20342.8 | 0 | NA |

*Relative to the model with the highest cross-validation likelihood

**Supplementary Table 3: Phenotype distribution of replication cohort**

| | | CD POP | | CD pediatric | | UC POP | | UC pediatric | |
|---|---|---|---|---|---|---|---|---|---|
| | | 1,097 | | 1,356 | | 3,106 | | 623 | |
| **Demographics** | | | | | | | | | |
| **Gender** | Male | 370 | 37.00% | 776 | 57.20% | 1362 | 51.60% | 278 | 44.60% |
| | Female | 630 | 63.00% | 580 | 42.80% | 1277 | 48.40% | 345 | 55.40% |
| | Missing | 418 | 8.80% | 0 | 0.00% | 467 | 15.00% | 0 | 0.00% |
| **Age at diagnosis** | Median (Quartiles) | 26y (20-37) | | 12y (10-14) | | 33y (24-44) | | 12y (8-15) | |
| | < 17 (A1) | 117 | 11.10% | 1224 | 90.30% | 124 | 5.60% | 559 | 89.70% |
| | 17 - 40 (A2) | 721 | 68.30% | 131 | 9.70% | 1379 | 62.20% | 64 | 10.30% |
| | > 40 (A3) | 218 | 20.60% | 0 | 0.00% | 714 | 32.20% | 0 | 0.00% |
| | Missing | 41 | 3.70% | 1 | <0.1% | 889 | 28.60% | 0 | 0.00% |
| **Family History** | Yes | 216 | 27.90% | | | 374 | 20.60% | 2232 | |
| | No | 558 | 72.10% | | | 1445 | 79.40% | 8260 | |
| | Missing | 323 | 29.40% | 1356 | 100.00% | 1287 | 41.40% | 623 | 100.00% |
| **Smoking Status** | Smoker | 300 | 31.10% | | | 134 | 9.00% | | |
| | Ex-Smoker | 245 | 25.40% | | | 567 | 38.30% | | |
| | Non-Smoker | 419 | 43.50% | | | 781 | 52.70% | | |
| | Missing | 133 | 12.10% | 1356 | 100.00% | 1624 | 52.30% | 623 | 100.00% |
| **Phenotypes** | | | | | | | | | |
| **Disease Location** | Ileal (L1) | 301 | 30.80% | 227 | 18.50% | | | | |
| | Colorectal (L2) | 264 | 27.00% | 347 | 28.30% | | | | |
| | Ileocolonic (L3) | 390 | 40.00% | 647 | 52.80% | | | | |
| | Other | 21 | 2.20% | 5 | 0.40% | | | | |
| | Upper GI (L4) | 93 | 14.10% | 332 | 31.10% | | | | |
| | Missing | 121 | 11.00% | 130 | 9.60% | | | | |
| **Disease Extent** | Proctitis (E1) | | | | | 386 | 16.70% | 0 | 0.00% |
| | Left-Sided (E2) | | | | | 921 | 39.90% | 144 | 30.40% |
| | Extensive (E3) | | | | | 994 | 43.10% | 329 | 69.60% |
| | Other | | | | | 7 | 0.30% | 0 | 0.00% |
| | Missing | | | | | 798 | 25.70% | 150 | 24.00% |
| **Disease Behaviour** | Inflammatory (B1) | 379 | 37.00% | 664 | 69.10% | | | | |
| | Stricturing (B2) | 355 | 34.60% | 133 | 13.80% | | | | |
| | Penetrating (B3) | 291 | 28.40% | 164 | 17.10% | | | | |
| | Missing | 72 | 6.60% | 395 | 29.10% | | | | |
| **Surgery** | Yes | 617 | 65.10% | | | 312 | 16.20% | | |
| | No | 331 | 34.90% | | | 1616 | 83.80% | | |
| | Missing | 149 | 13.60% | 1356 | 100.00% | 1178 | 37.90% | 623 | 100.00% |

**Supplementary Table 4: Suggestive ($p<1\times10^{-05}$) genetic association results for all studied phenotypes**

See Appendix B

**Supplementary Table 5: Genotype-phenotype association results for the 163 known IBD loci**

See Appendix B

**Supplementary Table 6: MHC associations for disease susceptibility and disease sub-phenotypes**

See Appendix B

**Supplementary Table 7: Predictive accuracy of risk scores based on SNPs and HLA types, assessed by cross-validation*.**

| Model | CD vs UC | Ileal CD vs Colonic CD |
|---|---|---|
| **SNP AUC****  | 0.601 (0.568 - 0.633) | 0.566 (0.532 - 0.598) |
| **HLA types AUC** | 0.589 (0.556 - 0.621) | 0.611 (0.579 - 0.643) |
| **SNP+HLA type AUC** | 0.622 (0.589 - 0.653) | 0.625 (0.593 - 0.657) |
| **HLA+SNP vs SNP P-value** | 0.026 | $2.20 \times 10^{-09}$ |
| **HLA+SNP vs HLA P-value** | 0.003 | 0.6715 |

*Models are trained in the non-UK data, and are validated in patients form UK by classifying purely ileal vs purely colonic data. P-values for improvement in AUC are calculated by testing for an excess of positive rank changes in ileal samples over colonic samples, via a Wilcoxon Rank Sum test. Numbers in brackets are 95% confidence intervals.

**AUC is for Area Under the (ROC) Curve. This is also the probability for a patient taken at random in the non-reference group to have a higher risk score than a patient taken at random in the reference group.

**Supplementary Table 8: Clinical characteristics for CD patients with low and high CDvsUC risk scores**

| Phenotype | | CDvsUC low (log ≤ -2), n=82 | CDvsUC high (log > 3), n=107 |
|---|---|---|---|
| **Disease location** | Ileal (L1) | 9 (15.3%) | 39 (36.4%) |
| | Ileocolonic (L3) | 21 (35.6%) | 33 (30.8%) |
| | Colorectal (L2) | 29 (49.2%) | 5 (4.7%) |
| | Missing | 23 (28.0%) | 14 (13.1%) |
| **Disease behaviour** | Inflammatory (B1) | 46 (77.9%) | 31 (40.3%) |
| | Stricturing (B2) | 7 (11.9%) | 26 (33.8%) |
| | Fistulating (B3) | 6 (10.2%) | 20 (25.9%) |
| | Missing | 23 (28.0%) | 30 (28.0%) |

The number and percentage of patients for each sub-phenotype is given.

**Supplementary Table 9: Review of diagnosis and genetic risk score**

| Original diagnosis | Feedback diagnosis | Non-outlier score | Outlier score |
|---|---|---|---|
| **CD** | CD | 59 (90.8%) | 48 (70.6%) |
| | UC/doubt | 6 (9.2%) | 20 (29.4%) |
| **UC** | UC | 28 (93.3%) | 23 (79.3%) |
| | CD/doubt | 2 (6.7%) | 6 (20.7%) |

The number and percentage of patients where the feedback diagnosis was the same or different from the original diagnosis is given for patients with an outlying CDvsUC risk score, or with a non-outlying CDvsUC risk score.

**Supplementary Figures**

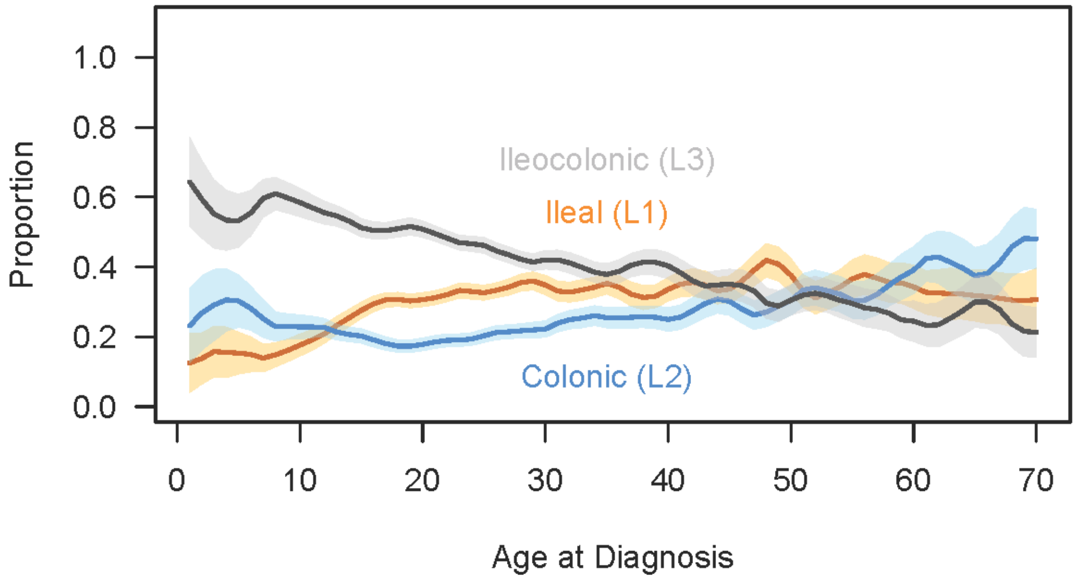


**Supplementary Figure 1: CD disease location versus age at diagnosis.** Observed distribution of disease location versus age at diagnosis is shown as smoothed proportions, with 95% confidence band. Ileal, ileocolonic and colonic location are shown respectively in orange, gray and blue. These are not corrected for disease duration, which is correlated to age at diagnosis.

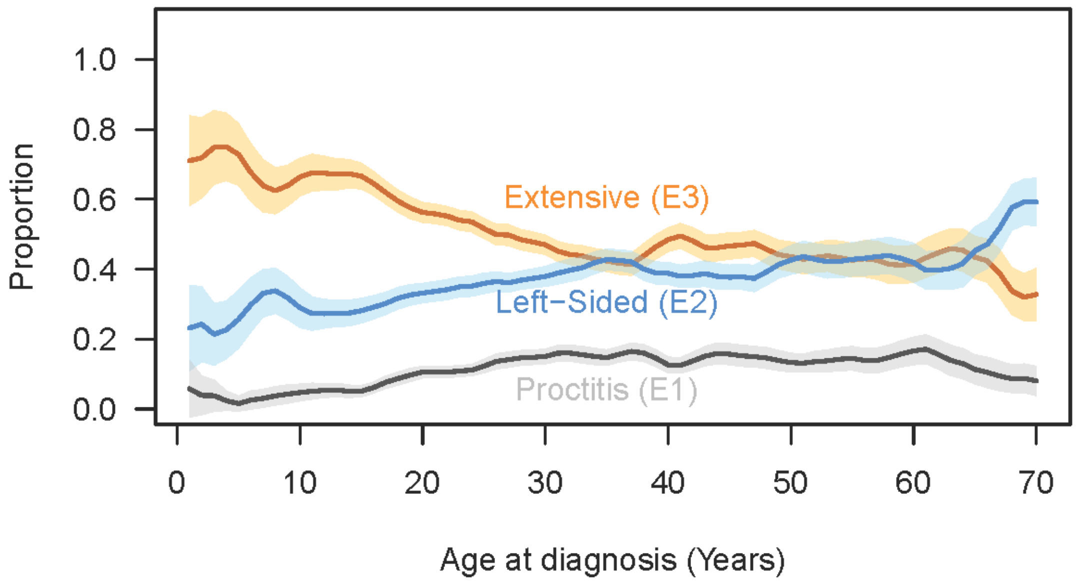


**Supplementary Figure 2: UC disease extent versus age at diagnosis.** Observed distribution of disease extent versus age at diagnosis is shown as smoothed proportions, with 95% confidence band. Extensive, left-sided and proctitis are shown respectively in orange, blue and gray. These are not corrected for disease duration, which is correlated to age at diagnosis.

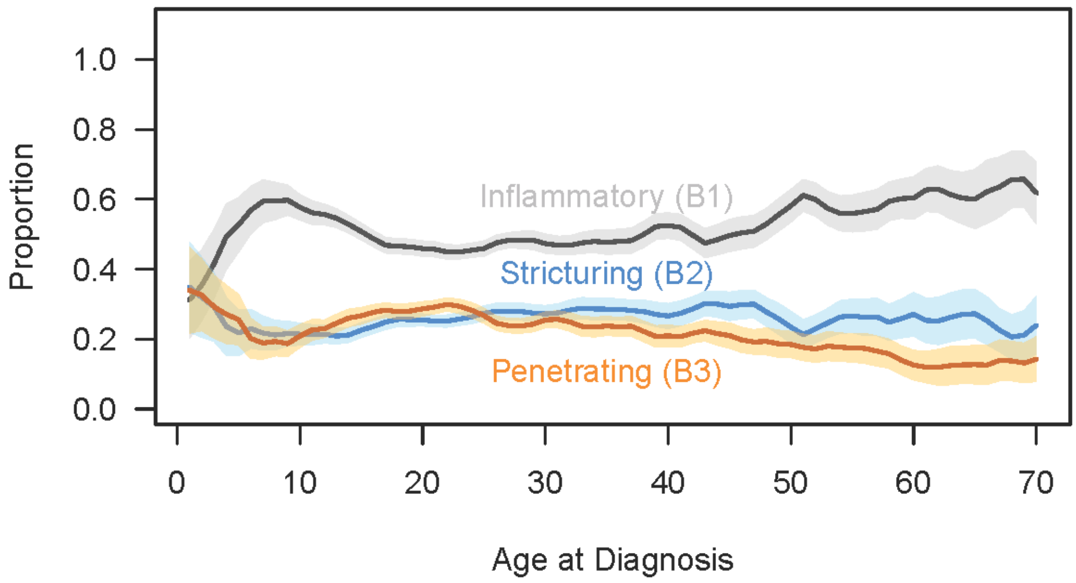


**Supplementary Figure 3: CD disease behaviour versus age at diagnosis.** Observed distribution of disease behaviour versus age at diagnosis is shown as smoothed proportions, with 95% confidence band. Inflammatory, stricturing and penetrating disease are shown respectively in gray, blue and orange. These are not corrected for disease duration, which is correlated to age at diagnosis.

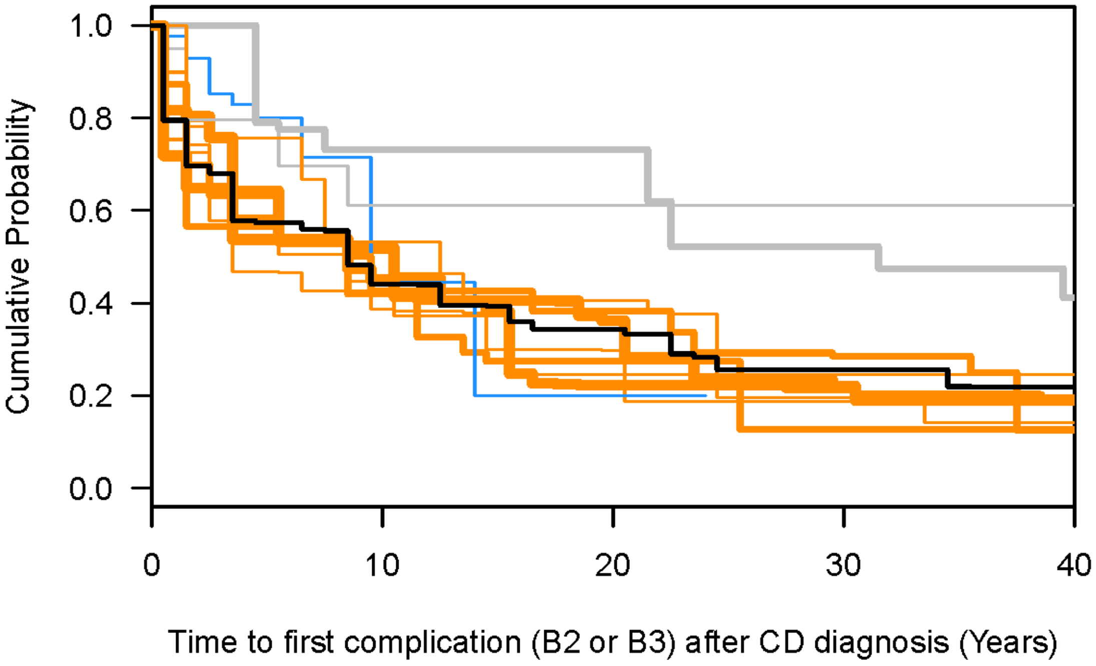


**Supplementary Figure 4: Time to first complication after CD diagnosis.** Black line represents the survival curve from the combined centres. Orange lines represent the secondary and tertiary centres. Gray lines represent the population based centres from Scandinavia. The blue line represents the EO-IBD (early-onset) cohort. The width of the lines is proportional to the sample size.

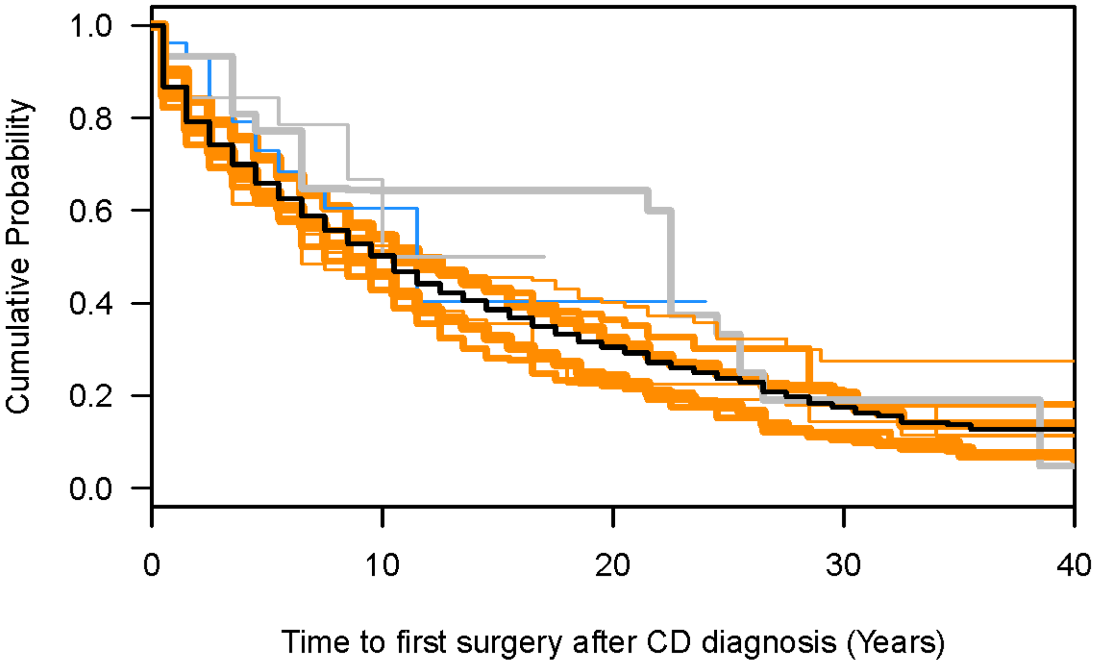


**Supplementary Figure 5: Time to first surgery after CD diagnosis.** Black line represents the survival curve from the combined centres. Orange lines represent the secondary and tertiary centres. Gray lines represent the population based centres from Scandinavia. The blue line represents the EO-IBD (early-onset) cohort. The width of the lines is proportional to the sample size.

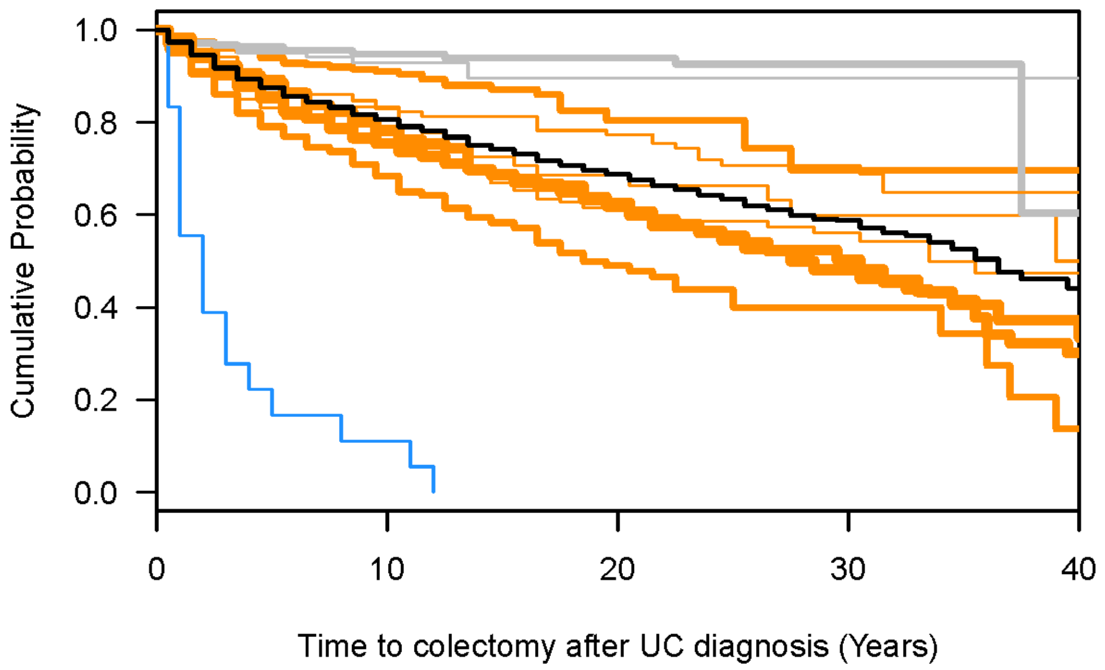


**Supplementary Figure 6: Time to colectomy after UC diagnosis.** Black line represents the survival curve from the combined centres. Orange lines represent the secondary and tertiary centres. Gray lines represent the population based centres from Scandinavia. The blue line represents the EO-IBD (early-onset) cohort. The width of the lines is proportional to the sample size.

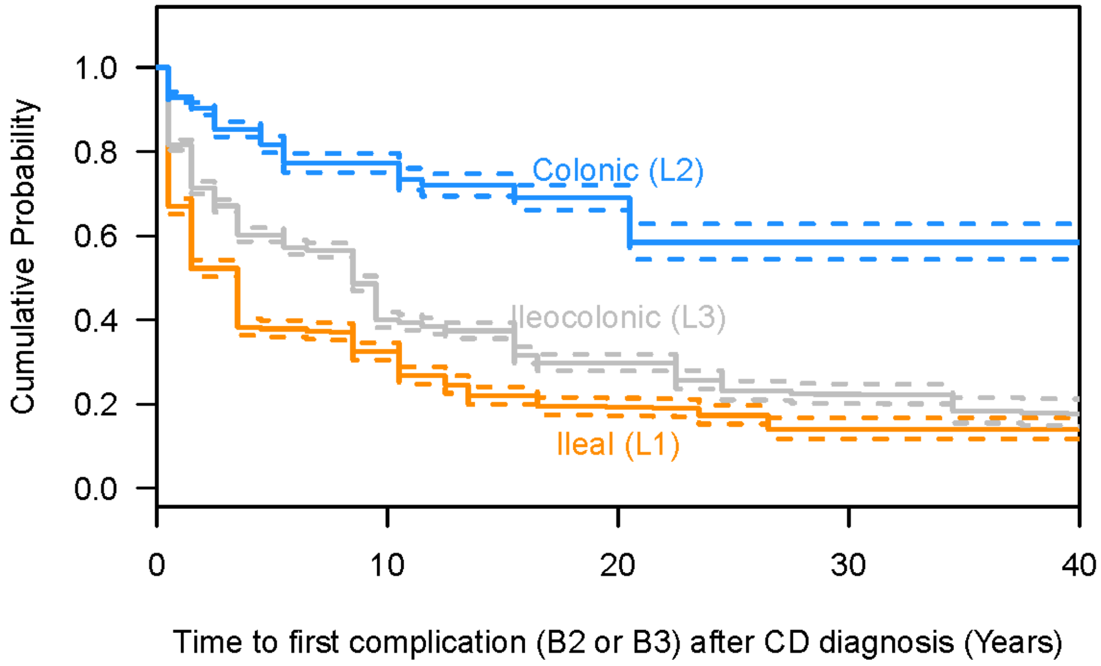


**Supplementary Figure 7: Time to first complication (B2 or B3) after CD diagnosis per disease location.** Kaplan Meier curves with 95% confidence intervals are plotted, conditional on disease location at last review; orange is for L1 – ileal, blue for L2 – colonic and gray for L3 – ileocolonic.

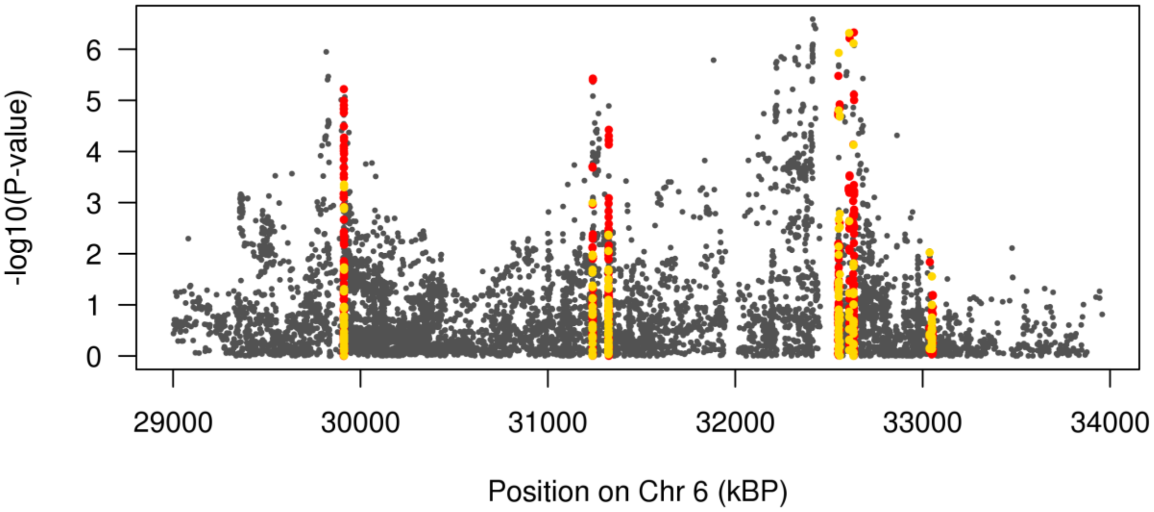


**Supplementary Figure 8: MHC region plot for CD age at diagnosis.** Evidence for association is shown as -$\log_{10}$(p-values) (y-axis). SNP variants are represented in gray, HLA alleles in yellow and HLA amino acid variants in red.

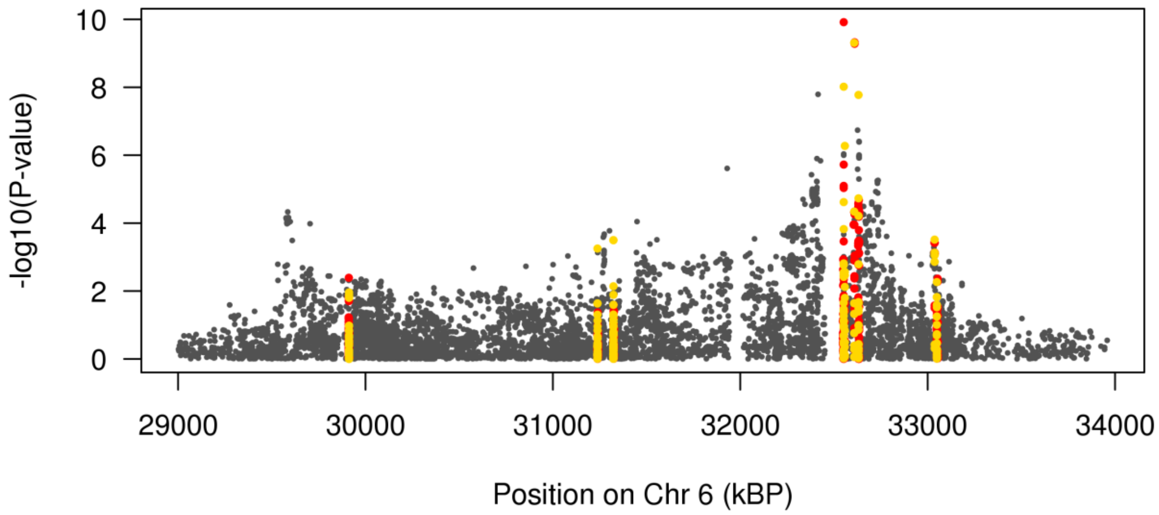


**Supplementary Figure 9: MHC region plot for UC age at diagnosis.** Evidence for association is shown as -$\log_{10}$(p-values) (y-axis). SNP variants are represented in gray, HLA alleles in yellow and HLA amino acid variants in red.

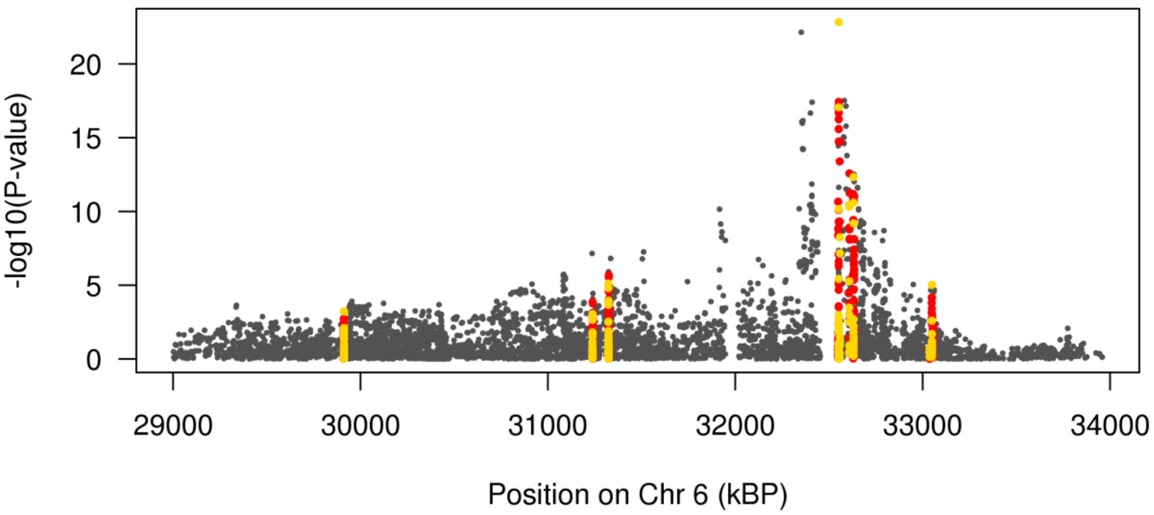


**Supplementary Figure 10: MHC region plot for CD location.** Evidence for association is shown as -$\log_{10}$(p-values) (y-axis). SNP variants are represented in gray, HLA alleles in yellow and HLA amino acid variants in red.

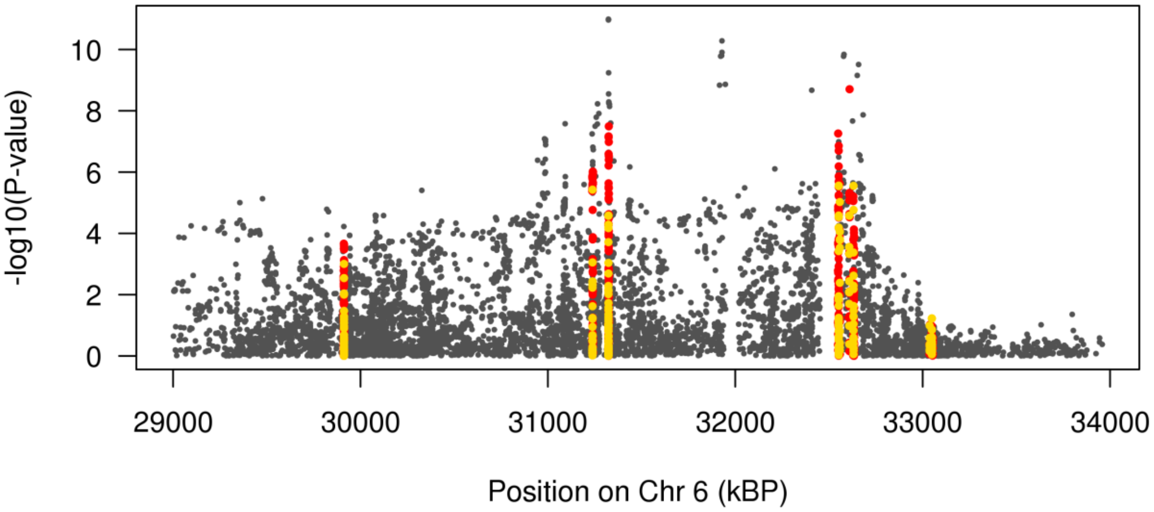


**Supplementary Figure 11: MHC region plot for CD behaviour.** Evidence for association is shown as -$\log_{10}$(p-values) (y-axis). SNP variants are represented in gray, HLA alleles in yellow and HLA amino acid variants in red.

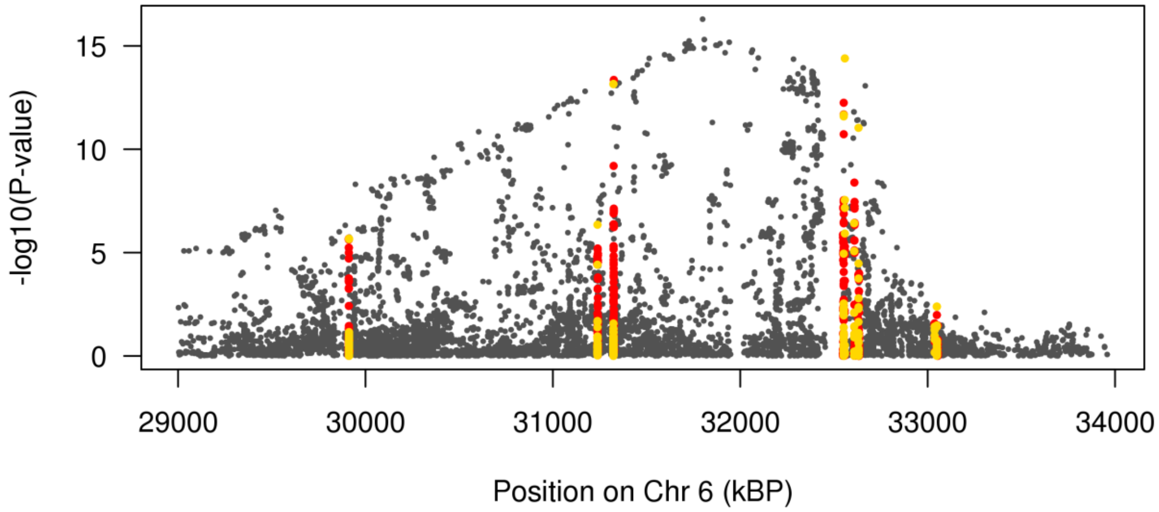


**Supplementary Figure 12: MHC region plot for UC extent.** Evidence for association is shown as -$\log_{10}$(p-values) (y-axis). SNP variants are represented in gray, HLA alleles in yellow and HLA amino acid variants in red.

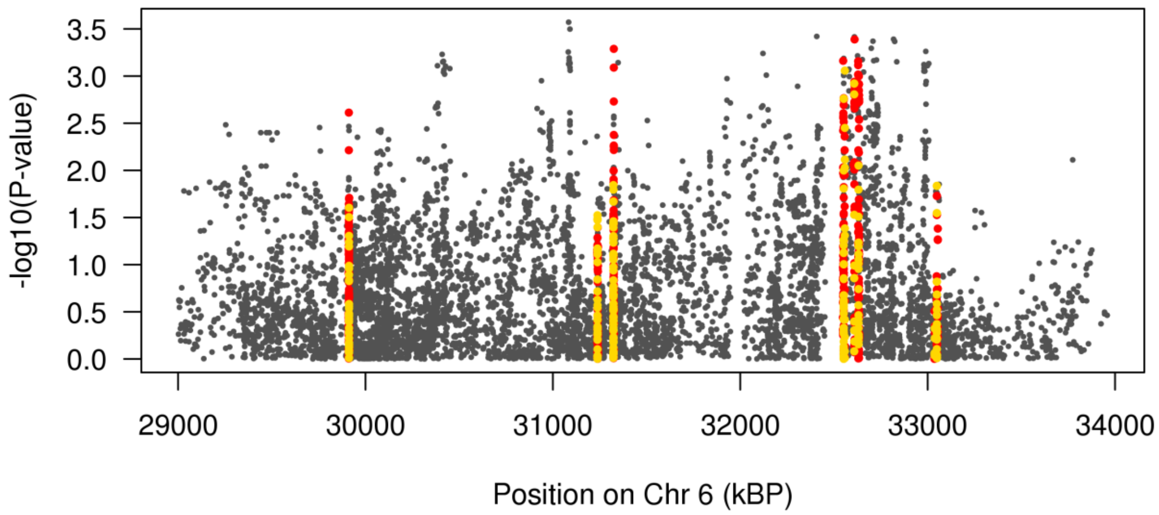


**Supplementary Figure 13: MHC region plot for CD surgery.** Evidence for association is shown as -$\log_{10}$(p-values) (y-axis). SNP variants are represented in gray, HLA alleles in yellow and HLA amino acid variants in red.

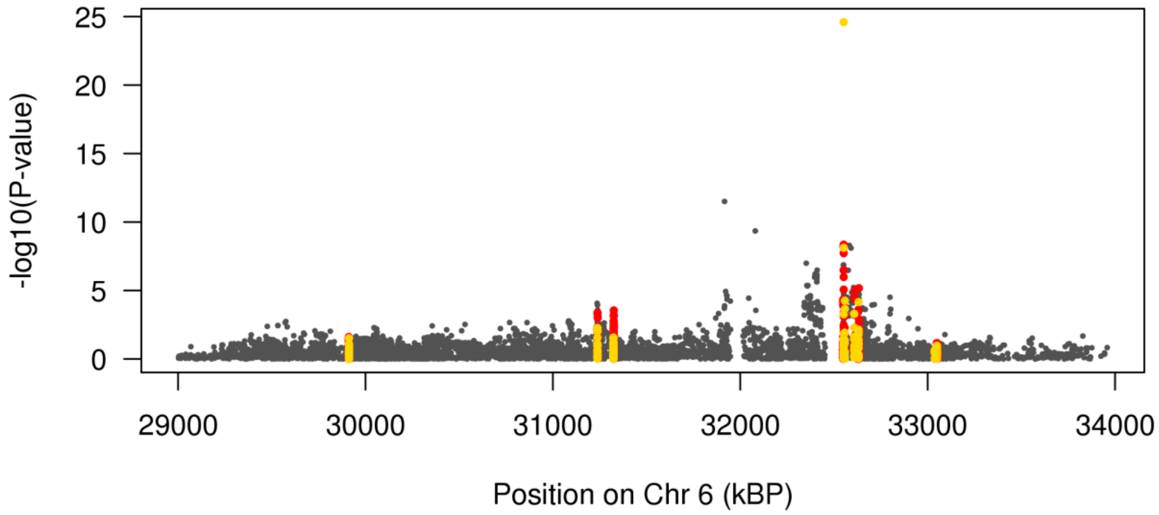


**Supplementary Figure 14: MHC region plot for UC colectomy.** Evidence for association is shown as -$\log_{10}$(p-values) (y-axis). SNP variants are represented in gray, HLA alleles in yellow and HLA amino acid variants in red.

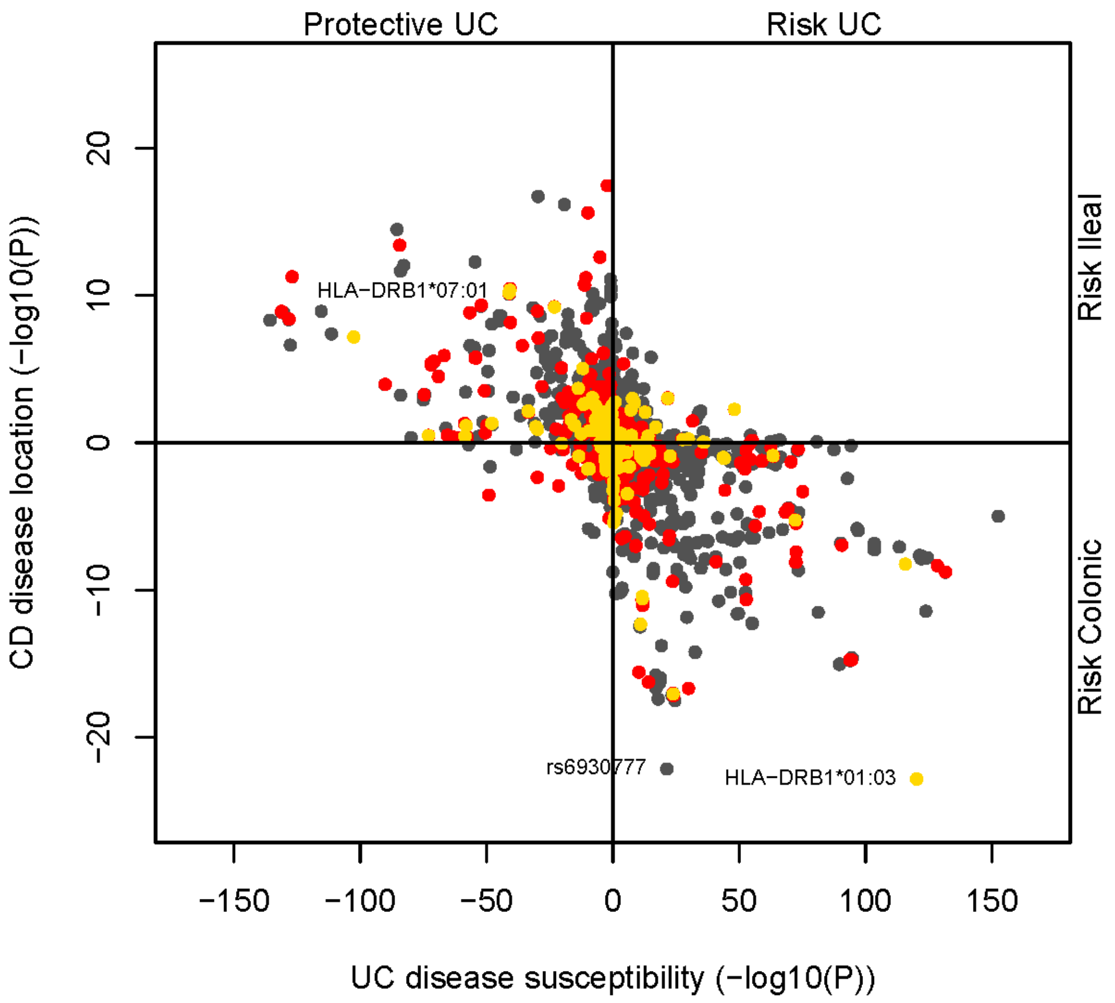


**Supplementary Figure 15: MHC association for CD disease location versus UC susceptibility.** Evidence of association is shown as $\pm\log_{10}$(p-values) for disease susceptibility in UC (x-axis) and disease location in CD (y-axis). The direction of the axis represents the direction of effect. For disease susceptibility (x-axis) risk alleles are represented on the positive (right) side, while protective alleles are shown on the negative (left) side. For disease location, alleles increasing risk to ileal location are on the positive (upper) part of the plot, while alleles increasing risk of colonic location are on the negative (lower) part of the plot. We can see many variants associated to UC risk are also associated to colonic location in CD, including HLA-DRB1*01:03.

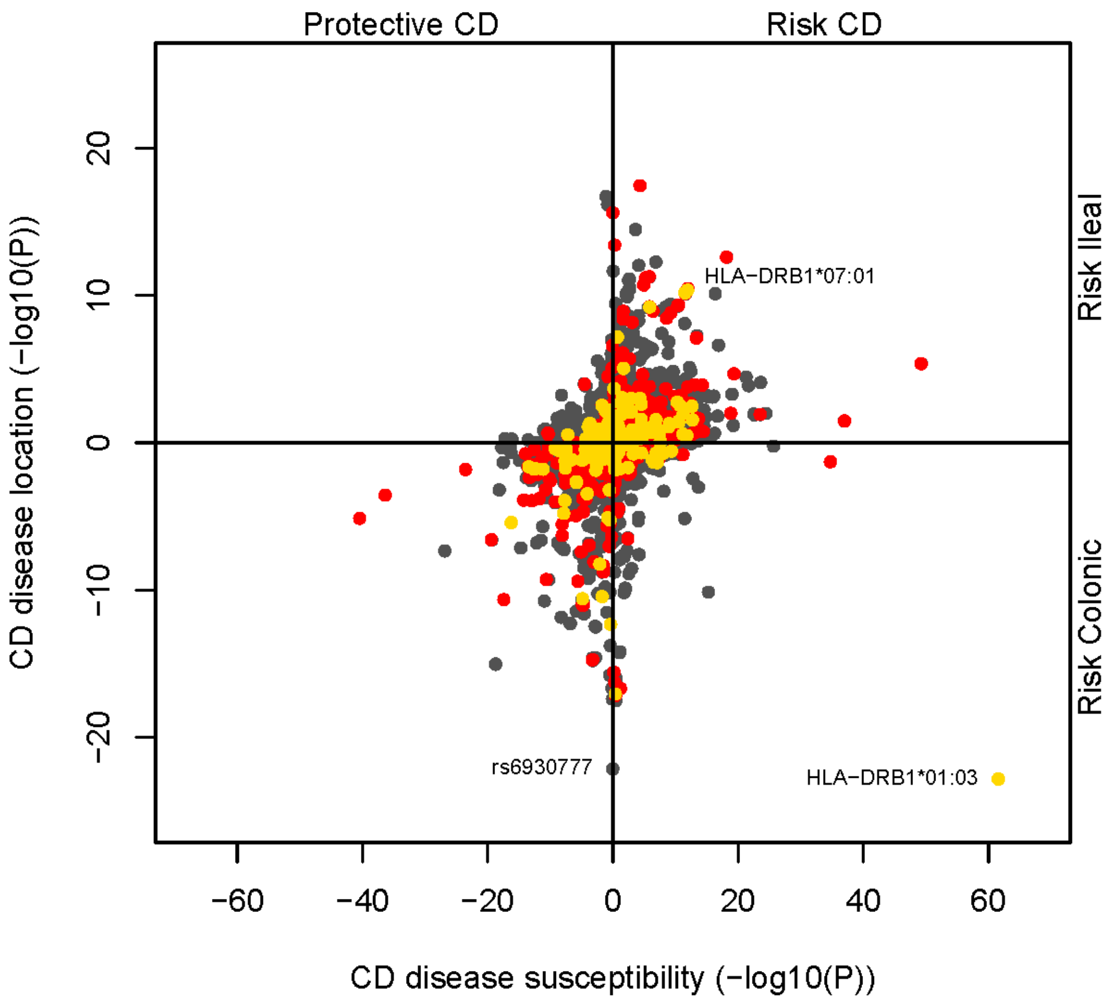


**Supplementary Figure 16: MHC association for CD disease location versus CD susceptibility.** Evidence of association is shown as $\pm\log_{10}$(p-values) for disease susceptibility in CD (x-axis) and disease location in CD (y-axis). The direction of the axis represents the direction of effect. For disease susceptibility (x-axis) risk alleles are represented on the positive (right) side, while protective alleles are shown on the negative (left) side. For disease location, alleles increasing risk to ileal location are on the positive (upper) part of the plot, while alleles increasing risk of colonic location are on the negative (lower) part of the plot. We can variants associated to CD risk are mostly associated to ileal location, a notable exception being HLA-DRB1*01:03.

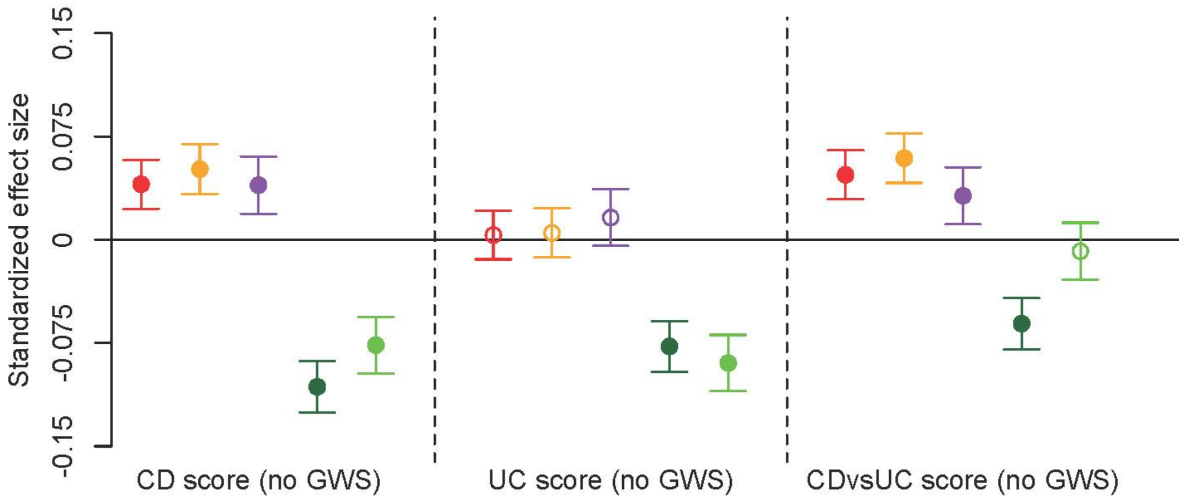


**Supplementary Figure 17: Effect sizes of genetic risk scores (GRS) for disease location, disease behavior and age at diagnosis including 159 susceptibility loci (with *NOD2*, MHC and *MST1* removed).** Effect sizes are calculated by linear regression on a standardized scale as in Figure 2B, and error bars depict 95% confidence intervals. Filled circles show parameters that differ significantly from zero after correcting for multiple testing (i.e. $p < 0.003$).

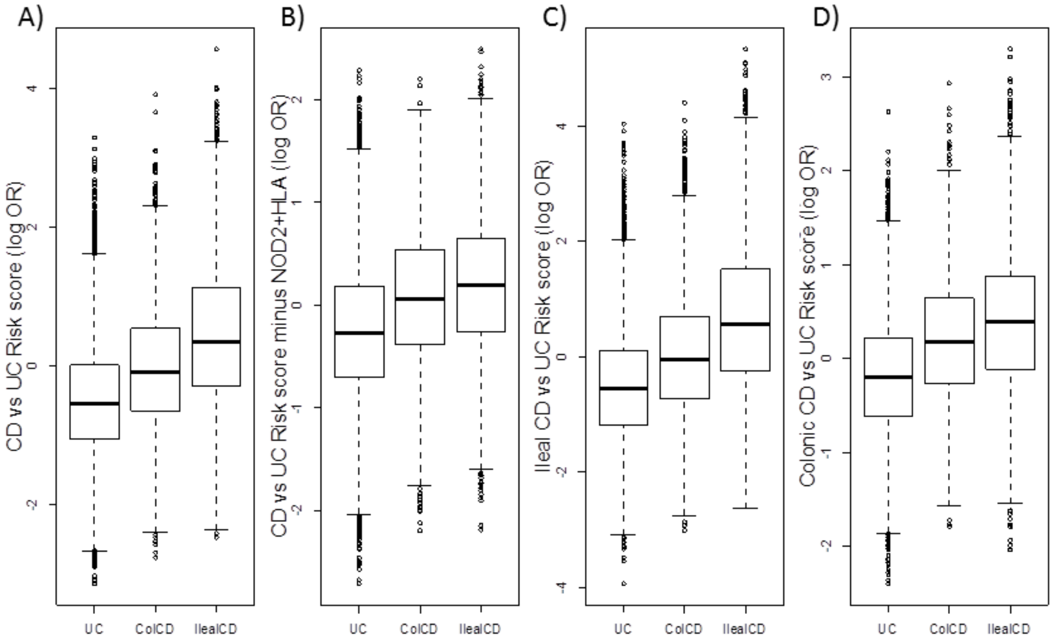


**Supplementary Figure 18: Consistent positioning of purely colonic CD as intermediate between purely ileal CD and UC on four different risk scores.** A) CD vs UC score, B) CD vs UC score with HLA and NOD2 removed, C) a score designed to seperate ileal CD from UC, D) a score designed to seperate colonic CD from UC.

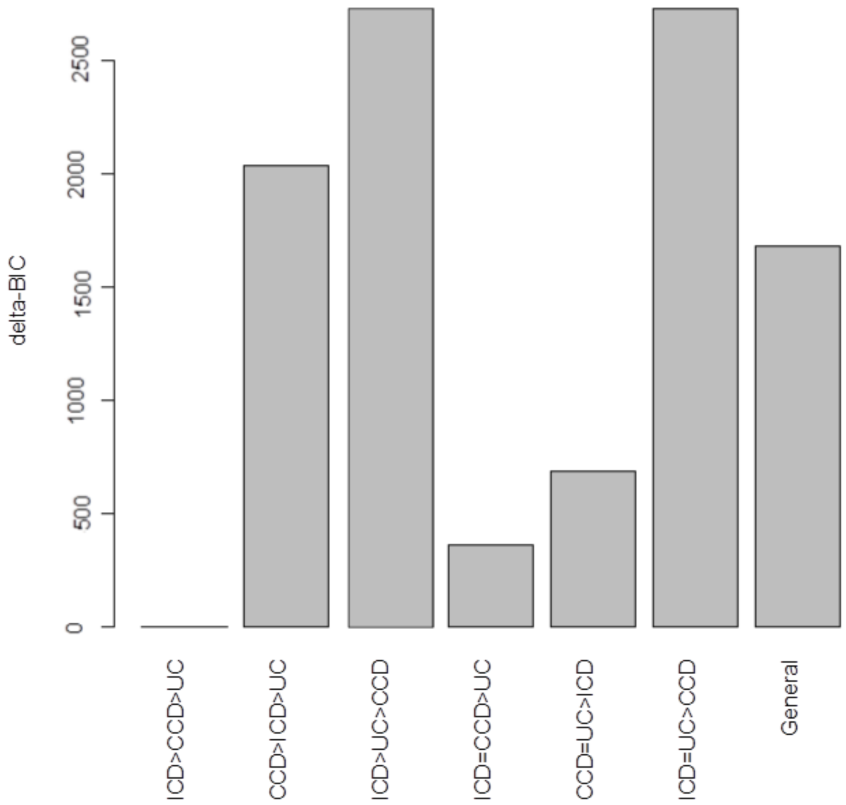


**Supplementary Figure 19: BIC-based phenotype model selection for UC, purely colonic CD (CCD) and purely ileal CD (ICD).** The ordinal model (first bar), corresponding to colonic CD as intermediate between ileal CD and UC, has the smallest delta-BIC, significantly smaller than the classical model (colonic CD and ileal CD as a single phenotype, distinct from UC, the fourth bar).

**References**


1. Shah TS, Liu JZ, Floyd JA, et al. optiCall: a robust genotype-calling algorithm for rare, low-frequency and common variants. *Bioinformatics* 2012; **28**(12): 1598-603.
2. Purcell S, Neale B, Todd-Brown K, et al. PLINK: a tool set for whole-genome association and population-based linkage analyses. *Am J Hum Genet* 2007; **81**(3): 559-75.
3. Price AL, Weale ME, Patterson N, et al. Long-range LD can confound genome scans in admixed populations. *Am J Hum Genet* 2008; **83**(1): 132-5; author reply 5-9.
4. Morris JA, Randall JC, Maller JB, Barrett JC. Evoker: a visualization tool for genotype intensity data. *Bioinformatics* 2010; **26**(14): 1786-7.
5. Jia X, Han B, Onengut-Gumuscu S, et al. Imputing amino acid polymorphisms in human leukocyte antigens. *PLoS One* 2013; **8**(6): e64683.
6. Goyette P, Boucher G, Mallon D, et al. High-density mapping of the MHC identifies a shared role for HLA-DRB1*01:03 in inflammatory bowel diseases and heterozygous advantage in ulcerative colitis. *Nat Genet* 2015.
7. Klein JP, Moeschberger ML. Survival Analysis: Techniques for Censored and Truncated Data: Springer; 2003.
8. Jostins L, Ripke S, Weersma RK, et al. Host-microbe interactions have shaped the genetic architecture of inflammatory bowel disease. *Nature* 2012; **491**(7422): 119-24.
9. Hosmer DW, Lemeshow S. Applied Logistic Regression: Wiley; 2004.
10. Schwarz G. Estimating Dimension of a Model. *Ann Stat* 1978; **6**(2): 461-4.
11. Ando T. Bayesian Model Selection and Statistical Modeling: Taylor & Francis; 2010.
12. Burnham KP, Anderson DR. Model Selection and Multimodel Inference: A Practical Information-Theoretic Approach: Springer; 2002.
13. Jostins L, Levine AP, Barrett JC. Using genetic prediction from known complex disease Loci to guide the design of next-generation sequencing experiments. *PLoS One* 2013; **8**(10): e76328.

**International IBD Genetics Consortium list of participants and affiliation (alphabetically)**

*The names and affiliations listed were checked and adapted if necessary*

| FIRST NAME | MIDDLE NAME | LAST NAME | AFFILIATION(S) |
|---|---|---|---|
| Clara | | Abraham | Section of Digestive Diseases, Department of Internal Medicine, Yale School of Medicine, NewHaven, Connecticut, USA |
| Jean-Paul | | Achkar | (1) Department of Gastroenterology and Hepatology, Digestive Disease Institute, Cleveland Clinic, Cleveland, Ohio, USA<br>(2) Department of Pathobiology, Lerner Research Institute, Cleveland Clinic, Cleveland, Ohio, USA |
| Tariq | | Ahmad | Peninsula College of Medicine and Dentistry, Exeter, UK |
| Leila | | Amininejad | (1) Department of Gastroenterology, Erasmus Hospital, Brussels, Belgium<br>(2) Department of Gastroenterology, Free University of Brussels, Brussels, Belgium |
| Ashwin | N | Ananthakrishnan | (1) Gastroenterology Unit, Massachusetts General Hospital, Harvard Medical School, Boston, Massachusetts, USA<br>(2) Division of Medical Sciences, Harvard Medical School, Boston, Massachusetts, USA |
| Vibeke | | Andersen | (1) Medical Department, Viborg Regional Hospital, Viborg, Denmark<br>(2) Organ Center, Hospital of Southern Jutland Aabenraa, Aabenraa, Denmark |
| Carl | A | Anderson | Wellcome Trust Sanger Institute, Wellcome Trust Genome Campus, Hinxton (Cambridge), UK |
| Jane | M | Andrews | Inflammatory Bowel Disease Service, Department of Gastroenterology and Hepatology, Royal Adelaide Hospital, Adelaide, Australia |
| Vito | | Annese | (1) Unit of Gastroenterology, Istituto di Ricovero e Cura a Carattere Scientifico-Casa Sollievo della Sofferenza (IRCCS-CSS) Hospital, San Giovanni Rotondo, Italy<br>(2) Unit of Gastroenterology SOD2, Azienda Ospedaliero Universitaria (AOU) Careggi, Florence, Italy |
| Guy | | Aumais | (1) Department of Gastroenterology, Hôpital Maisonneuve-Rosemont, Montréal, Québec, Canada<br>(2) Faculté de Médecine, Université de Montréal, Montréal, Québec, Canada |
| Leonard | | Baidoo | Division of Gastroenterology, Hepatology and Nutrition, Department of Medicine, University of Pittsburgh School of Medicine, Pittsburgh, Pennsylvania, USA |
| Robert | N | Baldassano | Center for Applied Genomics, The Children's Hospital of Philadelphia, Philadelphia, Pennsylvania, USA |
| Peter | A | Bampton | Department of Gastroenterology and Hepatology, Flinders Medical Centre and School of Medicine, Flinders University, Adelaide, Australia |
| Murray | | Barclay | Department of Medicine, University of Otago, Christchurch, New Zealand |
| Jeffrey | C | Barrett | Wellcome Trust Sanger Institute, Wellcome Trust Genome Campus, Hinxton (Cambridge), UK |
| Theodore | M | Bayless | Meyerhoff Inflammatory Bowel Disease Center, Department of medicine, Johns Hopkins University School of Medicine, |

| | | | |
|---|---|---|---|
| | | | Baltimore, Maryland, USA |
| Johannes | | Bethge | Department for General Internal Medicine, Christian-Albrechts-University, Kiel, Germany |
| Joshua | C | Bis | Cardiovascular Health Research Unit, University of Washington, Seattle, Washington, USA |
| Alain | | Bitton | Division of Gastroenterology, Royal Victoria Hospital, Montréal, Québec, Canada |
| Gabrielle | | Boucher | Research Center, Montreal Heart Institute, Montréal, Québec, Canada |
| Stephan | | Brand | Department of Medicine II, Ludwig-Maximilians-University Hospital Munich-Grosshadern, Munich, Germany |
| Berenice | | Brandt | Department for General Internal Medicine, Christian-Albrechts-University, Kiel, Germany |
| Steven | R | Brant | Meyerhoff Inflammatory Bowel Disease Center, Department of medicine, Johns Hopkins University School of Medicine, Baltimore, Maryland, USA |
| Carsten | | Büning | Department of Gastroenterology, Campus Charité Mitte, Universitatsmedizin Berlin, Berlin, Germany |
| Angela | | Chew | (1) IBD unit, Fremantle Hospital, Fremantle, Australia<br>(2) School of Medicine and Pharmacology, University of Western Australia, Fremantle, Australia |
| Judy | H | Cho | Department of Genetics, Yale School of Medicine, New Haven, Connecticut, USA |
| Isabelle | | Cleynen | (1) Wellcome Trust Sanger Institute, Wellcome Trust Genome Campus, Hinxton (Cambridge), UK<br>(2) Department of Clinical and experimental medicine, Translational Research in GastroIntestinal Disorders (TARGID), Katholieke Universiteit (KU) Leuven, Leuven, Belgium |
| Ariella | | Cohain | Department of Genetics and Genomic Sciences, Mount Sinai School of Medicine, New York, New York, USA |
| Anthony | | Croft | Inflammatory Bowel Diseases, Genetics and Computational Biology, Queensland Institute of Medical Research, Brisbane, Australia |
| Mark | J | Daly | (1) Analytic and Translational Genetics Unit, Massachusetts General Hospital, Harvard Medical School, Boston, Massachusetts, USA<br>(2) Broad Institute of MIT and Harvard, Cambridge, Massachusetts, USA |
| Mauro | | D'Amato | Department of Biosciences and Nutrition, Karolinska Institutet, Stockholm, Sweden |
| Silvio | | Danese | IBD Center, Department of Gastroenterology, Istituto Clinico Humanitas, Milan, Italy |
| Dirk | | De Jong | Department of Gastroenterology and Hepatology, Radboud University Nijmegen Medical Centre, Nijmegen, The Netherlands |
| Martine | | De Vos | Department of Hepatology and Gastroenterology, Ghent University Hospital, Ghent, Belgium |
| Goda | | Denapiene | Center of hepatology, Gastroenterology and Dietetics, Vilnius University, Vilnius, Lithuania |
| Lee | A | Denson | Pediatric Gastroenterology, Cincinnati Children's Hospital Medical Center, Cincinnati, Ohio, USA |
| Kathy | L | Devaney | Gastroenterology Unit, Massachusetts General Hospital, Harvard Medical School, Boston, Massachusetts, USA |
| Olivier | | Dewit | Department of Gastroenterology, Université Catholique de Louvain (UCL) Cliniques Universitaires Saint-Luc, Brussels, Belgium |

| | | | |
|---|---|---|---|
| Renata | | D'Inca | Division of Gastroenterology, University Hospital Padua, Padua, Italy |
| Marla | | Dubinsky | Department of Pediatrics, Cedars Sinai Medical Center, Los Angeles, California, USA |
| Richard | H | Duerr | (1) Division of Gastroenterology, Hepatology and Nutrition, Department of Medicine, University of Pittsburgh School of Medicine, Pittsburgh, Pennsylvania, USA<br>(2) Department of Human Genetics, University of Pittsburgh Graduate School of Public Health, Pittsburgh, Pennsylvania, USA |
| Cathryn | | Edwards | Department of Gastroenterology, Torbay Hospital, Torbay, Devon, UK |
| David | | Ellinghaus | Institute of Clinical Molecular Biology, Christian-Albrechts-University, Kiel, Germany |
| Jonah | | Essers | (1) Center for Human Genetic Research, Massachusetts General Hospital, Harvard Medical School, Boston, Massachusetts, USA<br>(2) Pediatrics, Harvard Medical School, Boston, Massachusetts, USA |
| Lynnette | R | Ferguson | Faculty of Medical & Health Sciences, School of Medical Sciences, The University of Auckland, Auckland, New Zealand |
| Eleonora | A | Festen | Department of Gastroenterology and Hepatology, University Medical Center Groningen, Groningen, The Netherlands |
| Philip | | Fleshner | F.Widjaja Foundation Inflammatory Bowel and Immunobiology Research Institute, Cedars-Sinai Medical Center, Los Angeles, California, USA |
| Tim | | Florin | Department of Gastroenterology, Mater Health Services, Brisbane, Australia |
| Denis | | Franchimont | (1) Department of Gastroenterology, Erasmus Hospital, Brussels, Belgium<br>(2) Department of Gastroenterology, Free University of Brussels, Brussels, Belgium |
| Andre | | Franke | Institute of Clinical Molecular Biology, Christian-Albrechts-University, Kiel, Germany |
| Karin | | Fransen | Department of Genetics, University Medical Center Groningen, Groningen, The Netherlands |
| Richard | | Gearry | (1) Department of Medicine, University of Otago, Christchurch, New Zealand<br>(2) Department of Gastroenterology, Christchurch Hospital, Christchurch, New Zealand |
| Michel | | Georges | (1) Unit of Animal Genomics, Groupe Interdisciplinaire de Génoprotéomique Appliquée (GIGA-R) Research Center, University of Liege, Liege, Belgium<br>(2) Faculty of Veterinary Medicine, University of Liege, Liege, Belgium |
| Christian | | Gieger | Institute of Genetic Epidemiology, Helmholtz Zentrum München - German Research Center for Environmental Health, Neuherberg, Germany |
| Jürgen | | Glas | (1) Department of Preventive Dentistry and Periodontology, Ludwig-Maximilians-University, Munich, Germany<br>(2) Department of Medicine II, Ludwig-Maximilians-University Hospital, Munich-Grosshadern, Munich, Germany |
| Philippe | | Goyette | Research Center, Montreal Heart Institute, Montréal, Québec, Canada |
| Todd | | Green | (1) Center for Human Genetic Research, Massachusetts General Hospital, Harvard Medical School, Boston, Massachusetts, USA<br>(2) Broad Institute of MIT and Harvard, Cambridge, Massachusetts, USA |

| | | | |
|---|---|---|---|
| Anne | M | Griffiths | Gastroenterology, Hepatology and Nutrition, The Hospital for Sick Children, Toronto, Ontario, Canada |
| Stephen | L | Guthery | Department of Pediatrics, University of Utah School of Medicine, Salt Lake City, Utah, USA |
| Hakon | | Hakonarson | Center for Applied Genomics, The Children's Hospital of Philadelphia, Philadelphia, Pennsylvania, USA |
| Jonas | | Halfvarson | Department of Gastroenterology, Faculty of Medicine and Health, SE 701 82 Örebro University, Sweden |
| Katherine | | Hanigan | Inflammatory Bowel Diseases, Genetics and Computational Biology, Queensland Institute of Medical Research, Brisbane, Australia |
| Talin | | Haritunians | F.Widjaja Foundation Inflammatory Bowel and Immunobiology Research Institute, Cedars-Sinai Medical Center, Los Angeles, California, USA |
| Ailsa | | Hart | Department of Medicine, St Mark's Hospital, Harrow, Middlesex, UK |
| Chris | | Hawkey | Nottingham Digestive Diseases Centre, Queens Medical Centre, Nottingham, UK |
| Nicholas | K | Hayward | Genetic Epidemiology, Genetics and Computational Biology, Queensland Institute of Medical Research, Brisbane, Australia |
| Matija | | Hedl | Section of Digestive Diseases, Department of Internal Medicine, Yale School of Medicine, NewHaven, Connecticut, USA |
| Paul | | Henderson | (1) Paediatric Gastroenterology and Nutrition, Royal Hospital for Sick Children, Edinburgh, UK<br>(2) Child Life and Health, University of Edinburgh, Edinburgh, Scotland, UK |
| Xinli | | Hu | Division of Rheumatology Immunology and Allergy, Brigham and Women's Hospital, Boston, Massachusetts, USA |
| Hailiang | | Huang | (1) Analytic and Translational Genetics Unit, Massachusetts General Hospital, Harvard Medical School, Boston, Massachusetts, USA<br>(2) Broad Institute of MIT and Harvard, Cambridge, Massachusetts, USA |
| Ken | Y | Hui | Department of Genetics, Yale School of Medicine, New Haven, Connecticut, USA |
| Marcin | | Imielinski | Center for Applied Genomics, The Children's Hospital of Philadelphia, Philadelphia, Pennsylvania, USA |
| Andrew | | Ippoliti | F.Widjaja Foundation Inflammatory Bowel and Immunobiology Research Institute, Cedars-Sinai Medical Center, Los Angeles, California, USA |
| Laimas | | Jonaitis | Academy of Medicine, Lithuanian University of Health Sciences, Kaunas, Lithuania |
| Luke | | Jostins | (1) Wellcome Trust Centre for Human Genetics, University of Oxford, Headington, UK<br>(2) Christ Church, University of Oxford, St Aldates, UK |
| Tom | H | Karlsen | (1) Research Institute of Internal Medicine, Department of Transplantation Medicine, Division of Cancer, Surgery and Transplantation, Oslo University Hospital Rikshospitalet, Oslo, Norway<br>(2) Norwegian PSC Research Center, Department of Transplantation Medicine, Division of Cancer, Surgery and Transplantation, Oslo University Hospital Rikshospitalet, Oslo, Norway<br>(3) K.G. Jebsen Inflammation Research Centre, Institute of Clinical Medicine, University of Oslo, Oslo, Norway |
| Nicholas | A | Kennedy | Gastrointestinal Unit, Wester General Hospital University of Edinburgh, Edinburgh, UK |

| | | | |
|---|---|---|---|
| Mohammed Azam | | Khan | (1) Genetic Medicine, Manchester Academic Health Science Centre, Manchester, UK<br>(2) The Manchester Centre for Genomic Medicine, University of Manchester, Manchester, UK |
| Gediminas | | Kiudelis | Academy of Medicine, Lithuanian University of Health Sciences, Kaunas, Lithuania |
| Krupa | | Krishnaprasad | QIMR Berghofer Medical Research Institute, Royal Brisbane Hospital, Brisbane, Queensland, Australia |
| Subra | | Kugathasan | Department of Pediatrics, Emory University School of Medicine, Atlanta, Georgia, USA |
| Limas | | Kupcinskas | Department of Gastroenterology, Lithuanian University of Medicine, Kaunas, Lithuania |
| Anna | | Latiano | Unit of Gastroenterology, Istituto di Ricovero e Cura a Carattere Scientifico-Casa Sollievo della Sofferenza (IRCCS-CSS) Hospital, San Giovanni Rotondo, Italy |
| Debby | | Laukens | Department of Hepatology and Gastroenterology, Ghent University Hospital, Ghent, Belgium |
| Ian | C | Lawrance | (1) Centre for inflammatory Bowel Diseases, Saint John of God Hospital, Subiaco, WA<br>(2) School of Medicine and Pharmacology, University of Western Australia, Harry Perkins Institute for Medical Research, Murdoch, WA, Australia |
| James | C | Lee | Inflammatory Bowel Disease Research Group, Addenbrooke's Hospital, Cambridge, UK |
| Charlie | W | Lees | Gastrointestinal Unit, Wester General Hospital University of Edinburgh, Edinburgh, UK |
| Marcis | | Leja | Faculty of medicine, University of Latvia, Riga, Latvia |
| Johan Van | | Limbergen | Division of Pediatric Gastroenterology, Hepatology and Nutrition, Hospital for Sick Children, Toronto, Ontario, Canada |
| Paolo | | Lionetti | Dipartimento di Neuroscienze, Psicologia, Area del Farmaco e Salute del Bambino (NEUROFARBA), Università di Firenze SOD Gastroenterologia e Nutrizione Ospedale pediatrico Meyer, Firenze, Italy |
| Jimmy | Z | Liu | Wellcome Trust Sanger Institute, Wellcome Trust Genome Campus, Hinxton (Cambridge), UK |
| Edouard | | Louis | Division of Gastroenterology, University Hospital CHU of Liege, Liege, Belgium |
| Gillian | | Mahy | Department of Gastroenterology, The Townsville Hospital, Townsville, Australia |
| John | | Mansfield | Institute of Human Genetics, Newcastle University, Newcastle upon Tyne, UK |
| Dunecan | | Massey | Inflammatory Bowel Disease Research Group, Addenbrooke's Hospital, Cambridge, UK |
| Christopher | G | Mathew | (1) Department of Medical and Molecular Genetics, Guy's Hospital, London, UK<br>(2) Department of Medical and Molecular Genetics, King's College London School of Medicine, Guy's Hospital, London, UK |
| Dermot | PB | McGovern | F.Widjaja Foundation Inflammatory Bowel and Immunobiology Research Institute, Cedars-Sinai Medical Center, Los Angeles, California, USA |
| Raquel | | Milgrom | Inflammatory Bowel Disease Centre, Mount Sinai Hospital, Toronto, Ontario, Canada |
| Mitja | | Mitrovic | (1) Center for Human Molecular Genetics and Pharmacogenomics, Faculty of Medicine, University of Maribor, Maribor, Slovenia |

| | | | |
|---|---|---|---|
| | | | (2) Department of Genetics, University Medical Center Groningen, Groningen, The Netherlands |
| Grant | W | Montgomery | Genetic Epidemiology, Genetics and Computational Biology, Queensland Institute of Medical Research, Brisbane, Australia |
| Craig | | Mowat | Department of Medicine, Ninewells Hospital and Medical School, Dundee, UK |
| William | | Newman | (1) Genetic Medicine, Manchester Academic Health Science Centre, Manchester, UK<br>(2) The Manchester Centre for Genomic Medicine, University of Manchester, Manchester, UK |
| Aylwin | | Ng | (1) Center for Computational and Integrative Biology, Massachusetts General Hospital, Harvard Medical School, Boston, Massachusetts, USA<br>(2) Gastroenterology Unit, Massachusetts General Hospital, Harvard Medical School, Boston, Massachusetts, USA |
| Siew | C | Ng | Department of Medicine and Therapeutics, Institute of Digestive Disease, Chinese University of Hong Kong, Hong Kong |
| Sok Meng Evelyn | | Ng | Section of Digestive Diseases, Department of Internal Medicine, Yale School of Medicine, NewHaven, Connecticut, USA |
| Susanna | | Nikolaus | Department for General Internal Medicine, Christian-Albrechts-University, Kiel, Germany |
| Kaida | | Ning | Section of Digestive Diseases, Department of Internal Medicine, Yale School of Medicine, NewHaven, Connecticut, USA |
| Markus | | Nöthen | Department of Genomics Life & Brain Center, University Hospital Bonn, Bonn, Germany |
| Ioannis | | Oikonomou | Section of Digestive Diseases, Department of Internal Medicine, Yale School of Medicine, NewHaven, Connecticut, USA |
| Orazio | | Palmieri | Unit of Gastroenterology, Istituto di Ricovero e Cura a Carattere Scientifico-Casa Sollievo della Sofferenza (IRCCS-CSS) Hospital, San Giovanni Rotondo, Italy |
| Miles | | Parkes | Inflammatory Bowel Disease Research Group, Addenbrooke's Hospital, Cambridge, UK |
| Anne | | Phillips | Department of Medicine, Ninewells Hospital and Medical School, Dundee, UK |
| Cyriel | Y | Ponsioen | Department of Gastroenterology, Academic Medical Center, Amsterdam, The Netherlands |
| Urõs | | Potocnik | (1) Center for Human Molecular Genetics and Pharmacogenomics, Faculty of Medicine, University of Maribor, Maribor, Slovenia<br>(2) Faculty for Chemistry and Chemical Engineering, University of Maribor, Maribor, Slovenia |
| Natalie | J | Prescott | (1) Department of Medical and Molecular Genetics, Guy's Hospital, London, UK<br>(2) Department of Medical and Molecular Genetics, King's College London School of Medicine, Guy's Hospital, London, UK |
| Deborah | D | Proctor | Section of Digestive Diseases, Department of Medicine, Yale University, New Haven, Connecticut, USA |
| Graham | | Radford-Smith | (1) Inflammatory Bowel Diseases, Genetics and Computational Biology, Queensland Institute of Medical Research, Brisbane, Australia<br>(2) Department of Gastroenterology, Royal Brisbane and Womens Hospital, Brisbane, Australia |
| Jean-Francois | | Rahier | Department of Gastroenterology, Université Catholique de Louvain (UCL) Centre hospitalier (CHU) Mont-Godinne, Mont-Godinne, Belgium |

| | | | |
|---|---|---|---|
| Soumya | | Raychaudhuri | Division of Rheumatology Immunology and Allergy, Brigham and Women's Hospital, Boston, Massachusetts, USA |
| Miguel | | Regueiro | Division of Gastroenterology, Hepatology and Nutrition, Department of Medicine, University of Pittsburgh School of Medicine, Pittsburgh, Pennsylvania, USA |
| Florian | | Rieder | Department of Gastroenterology and Hepatology, Digestive Disease Institute, Cleveland Clinic, Cleveland, Ohio, USA |
| John | D | Rioux | (1) Research Center, Montreal Heart Institute, Montréal, Québec, Canada<br>(2) Faculté de Médecine, Université de Montréal, Montréal, Québec, Canada |
| Stephan | | Ripke | (1) Analytic and Translational Genetics Unit, Massachusetts General Hospital, Harvard Medical School, Boston, Massachusetts, USA<br>(2) Broad Institute of MIT and Harvard, Cambridge, Massachusetts, USA |
| Rebecca | | Roberts | Department of Medicine, University of Otago, Christchurch, New Zealand |
| Richard | K | Russell | Paediatric Gastroenterology and Nutrition, Royal Hospital for Sick Children, Edinburgh, UK |
| Jeremy | D | Sanderson | Department of Gastroenterology, Guy's & St Thomas' NHS Foundation Trust, St-Thomas Hospital, London, UK |
| Miquel | | Sans | Department of Digestive Diseases, Hospital Quiron Teknon, Barcelona, Spain |
| Jack | | Satsangi | Gastrointestinal Unit, Wester General Hospital University of Edinburgh, Edinburgh, UK |
| Eric | E | Schadt | Department of Genetics and Genomic Sciences, Mount Sinai School of Medicine, New York, New York, USA |
| Stefan | | Schreiber | (1) Institute of Clinical Molecular Biology, Christian-Albrechts-University, Kiel, Germany<br>(2) Department for General Internal Medicine, Christian-Albrechts-University, Kiel, Germany |
| L | Philip | Schumm | Department of Public Health Sciences, University of Chicago, Chicago, Illinois, USA |
| Regan | | Scott | Division of Gastroenterology, Hepatology and Nutrition, Department of Medicine, University of Pittsburgh School of Medicine, Pittsburgh, Pennsylvania, USA |
| Mark | | Seielstad | (1) Human Genetics, Genome Institute of Singapore, Singapore<br>(2) Institute for Human Genetics, University of California San Fransisco, San Francisco, California, USA |
| Yashoda | | Sharma | Section of Digestive Diseases, Department of Internal Medicine, Yale School of Medicine, NewHaven, Connecticut, USA |
| Mark | S | Silverberg | Inflammatory Bowel Disease Centre, Mount Sinai Hospital, Toronto, Ontario, Canada |
| Lisa | A | Simms | Inflammatory Bowel Diseases, Genetics and Computational Biology, Queensland Institute of Medical Research, Brisbane, Australia |
| Jurgita | | Skieceviciene | Academy of Medicine, Lithuanian University of Health Sciences, Kaunas, Lithuania |
| Sarah | L | Spain | Department of Medical and Molecular Genetics, King's College London School of Medicine, Guy's Hospital, London, UK |
| A. Hillary | | Steinhart | Inflammatory Bowel Disease Centre, Mount Sinai Hospital, Toronto, Ontario, Canada |
| Joanne | M | Stempak | Inflammatory Bowel Disease Centre, Mount Sinai Hospital, Toronto, Ontario, Canada |
| Laura | | Stronati | Department of Biology of Radiations and Human Health, Agenzia nazionale per le nuove tecnologie l'energia e lo sviluppo |

| | | | |
|---|---|---|---|
| | | | economico sostenibile (ENEA), Rome, Italy |
| Jurgita | | Sventoraityte | Department of Gastroenterology, Lithuanian University of Medicine, Kaunas, Lithuania |
| Stephan | R | Targan | F.Widjaja Foundation Inflammatory Bowel and Immunobiology Research Institute, Cedars-Sinai Medical Center, Los Angeles, California, USA |
| Kirstin | M | Taylor | Department of Gastroenterology, Guy's & St Thomas' NHS Foundation Trust, St-Thomas Hospital, London, UK |
| Anje | | ter Velde | Department of Gastroenterology, Academic Medical Center, Amsterdam, The Netherlands |
| Emilie | | Theatre | (1) Unit of Animal Genomics, Groupe Interdisciplinaire de Génoprotéomique Appliquée (GIGA-R) Research Center, University of Liege, Liege, Belgium<br>(2) Faculty of Veterinary Medicine, University of Liege, Liege, Belgium |
| Leif | | Torkvist | Department of Clinical Science Intervention and Technology, Karolinska Institutet, Stockholm, Sweden |
| Mark | | Tremelling | Gastroenterology & General Medicine, Norfolk and Norwich University Hospital, Norwich, UK |
| Andrea | | van der Meulen | Department of Gastroenterology, Leiden University Medical Center, Leiden, The Netherlands |
| Suzanne | | van Sommeren | Department of Gastroenterology and Hepatology, University Medical Center Groningen, Groningen, The Netherlands |
| Eric | | Vasiliauskas | F.Widjaja Foundation Inflammatory Bowel and Immunobiology Research Institute, Cedars-Sinai Medical Center, Los Angeles, California, USA |
| Severine | | Vermeire | (1) Division of Gastroenterology, University Hospital Gasthuisberg, Leuven, Belgium<br>(2) Department of Clinical and experimental medicine, Translational Research in GastroIntestinal Disorders (TARGID), Katholieke Universiteit (KU) Leuven, Leuven, Belgium |
| Hein | W | Verspaget | Department of Gastroenterology, Leiden University Medical Center, Leiden, The Netherlands |
| Thomas | | Walters | (1) Gastroenterology, Hepatology and Nutrition, The Hospital for Sick Children, Toronto, Ontario, Canada<br>(2) Faculty of medicine, University of Toronto, Toronto, Ontario, Canada |
| Kai | | Wang | Center for Applied Genomics, The Children's Hospital of Philadelphia, Philadelphia, Pennsylvania, USA |
| Ming-Hsi | | Wang | (1) Meyerhoff Inflammatory Bowel Disease Center, Department of medicine, Johns Hopkins University School of Medicine, Baltimore, Maryland, USA<br>(2) Department of Gastroenterology and Hepatology, Digestive Disease Institute, Cleveland Clinic, Cleveland, Ohio, USA |
| Rinse | K | Weersma | Department of Gastroenterology and Hepatology, University Medical Center Groningen, Groningen, The Netherlands |
| Zhi | | Wei | Department of Computer Science, New Jersey Institute of Technology, Newark, New Jersey, USA |
| David | | Whiteman | Molecular Epidemiology, Genetics and Computational Biology, Queensland Institute of Medical Research, Brisbane, Australia |
| Cisca | | Wijmenga | Department of Genetics, University Medical Center Groningen, Groningen, The Netherlands |
| David | C | Wilson | (1) Paediatric Gastroenterology and Nutrition, Royal Hospital for Sick Children, Edinburgh, UK<br>(2) Child Life and Health, University of Edinburgh, Edinburgh, Scotland, UK |

| | | | |
|---|---|---|---|
| Juliane | | Winkelmann | (1) Institute of Human Genetics, Technische Universität München, Munich, Germany<br>(2) Department of Neurology, Technische Universität München, Munich, Germany |
| Ramnik | J | Xavier | (1) Gastroenterology Unit, Massachusetts General Hospital, Harvard Medical School, Boston, Massachusetts, USA<br>(2) Broad Institute of MIT and Harvard, Cambridge, Massachusetts, USA |
| Sebastian | | Zeissig | Department for General Internal Medicine, Christian-Albrechts-University, Kiel, Germany |
| Bin | | Zhang | Department of Genetics and Genomic Sciences, Mount Sinai School of Medicine, New York, New York, USA |
| Clarence | K | Zhang | Department of Biostatistics, School of Public Health, Yale University, NewHaven, Connecticut, USA |
| Hu | | Zhang | (1) Department of Gastroenterology, West China Hospital, Chengdu, Sichuan, China<br>(2) State Key Laboratory of Biotherapy, Sichuan University West China University of Medical Sciences (WCUMS), Chengdu, Sichuan, China |
| Wei | | Zhang | Section of Digestive Diseases, Department of Internal Medicine, Yale School of Medicine, NewHaven, Connecticut, USA |
| Hongyu | | Zhao | Department of Biostatistics, School of Public Health, Yale University, NewHaven, Connecticut, USA |
| Zhen | Z | Zhao | Genetic Epidemiology, Genetics and Computational Biology, Queensland Institute of Medical Research, Brisbane, Australia |
| | | Australia and New Zealand IBDGC | |
| | | Belgium IBD Genetics Consortium | |
| | | Italian Group for IBD Genetic Consortium | |
| | | NIDDK Inflammatory Bowel Disease Genetics Consortium | |
| | | United Kingdom IBDGC | |
| | | Wellcome Trust Case Control Consortium | |
| | | Quebec IBD Genetics Consortium | |
